# Genome-resolved metagenomics of sugarcane vinasse bacteria

**DOI:** 10.1186/s13068-018-1036-9

**Published:** 2018-02-22

**Authors:** Noriko A. Cassman, Késia S. Lourenço, Janaína B. do Carmo, Heitor Cantarella, Eiko E. Kuramae

**Affiliations:** 1 Department of Microbial Ecology, Netherlands Institute of Ecology NIOO-KNAW, Wageningen, Netherlands; 2 Soils and Environmental Resources Center, Agronomic Institute of Campinas, P.O. Box 28, Campinas, SP 13012-970 Brazil; 3 Environmental Science Department, Federal University of São Carlos, Sorocaba, SP 18052-780 Brazil

**Keywords:** Sugarcane, Vinasse, Bioethanol, Sustainability, Genome binning, Metagenomics

## Abstract

**Background:**

The production of 1 L of ethanol from sugarcane generates up to 12 L of vinasse, which is a liquid waste containing an as-yet uncharacterized microbial assemblage. Most vinasse is destined for use as a fertilizer on the sugarcane fields because of the high organic and K content; however, increased N_2_O emissions have been observed when vinasse is co-applied with inorganic N fertilizers. Here we aimed to characterize the microbial assemblage of vinasse to determine the gene potential of vinasse microbes for contributing to negative environmental effects during fertirrigation and/or to the obstruction of bioethanol fermentation.

**Results:**

We measured chemical characteristics and extracted total DNA from six vinasse batches taken over 1.5 years from a bioethanol and sugar mill in Sao Paulo State. The vinasse microbial assemblage was characterized by low alpha diversity with 5–15 species across the six vinasses. The core genus was *Lactobacillus*. The top six represented bacterial genera across the samples were *Lactobacillus*, *Megasphaera* and *Mitsuokella* (Phylum Firmicutes, 35–97% of sample reads); *Arcobacter* and *Alcaligenes* (Phylum Proteobacteria, 0–40%); *Dysgonomonas* (Phylum Bacteroidetes, 0–53%); and *Bifidobacterium* (Phylum Actinobacteria, 0–18%). Potential genes for denitrification but not nitrification were identified in the vinasse metagenomes, with putative *nir*K and *nos*Z genes the most represented. Binning resulted in 38 large bins with between 36.0 and 99.3% completeness, and five small mobile element bins. Of the large bins, 53% could be classified at the phylum level as Firmicutes, 15% as Proteobacteria, 13% as unknown phyla, 13% as Bacteroidetes and 6% as Actinobacteria. The large bins spanned a range of potential denitrifiers; moreover, the genetic repertoires of all the large bins included the presence of genes involved in acetate, CO_2_, ethanol, H_2_O_2_, and lactose metabolism; for many of the large bins, genes related to the metabolism of mannitol, xylose, butyric acid, cellulose, sucrose, “3-hydroxy” fatty acids and antibiotic resistance were present based on the annotations. In total, 21 vinasse bacterial draft genomes were submitted to the genome repository.

**Conclusions:**

Identification of the gene repertoires of vinasse bacteria and assemblages supported the idea that organic carbon and nitrogen present in vinasse together with microbiological variation of vinasse might lead to varying patterns of N_2_O emissions during fertirrigation. Furthermore, we uncovered draft genomes of novel strains of known bioethanol contaminants, as well as draft genomes unknown at the phylum level. This study will aid efforts to improve bioethanol production efficiency and sugarcane agriculture sustainability.

**Electronic supplementary material:**

The online version of this article (10.1186/s13068-018-1036-9) contains supplementary material, which is available to authorized users.

## Background

Sao Paulo State contains a total of 5.7 million hectares of land planted with sugarcane. These fields supply the input for Brazil’s large bioethanol industry, which is the second largest producer of bioethanol worldwide (UNICA). Brazil has more than 300 sugarcane processing plants, including sugar mills (producing only sugar), mills with distillery plants (sugar and ethanol production), and independent distilleries (only ethanol production). In the 2013/2014 season, the total ethanol production was 13.9 thousand m^3^ (UNICA, 2013/2014 harvest). The major by-product of sugarcane ethanol production is vinasse; up to 12 L of vinasse is generated per liter of ethanol [1]. Sugarcane vinasse consists of water (about 93%) and organic compounds, and contains K, Ca and Mg, though the amount of these components depends on the characteristics of the input sugarcane and subsequent processing steps [2]. The major organic components of sugarcane vinasse are low molecular weight organic compounds, mainly glycerol, lactic acid, ethanol, and acetic acid [3]. In general, vinasse has a low pH of around 4 and high chemical oxygen demand of 100–500 g/L.

The large volumes of vinasse and its chemical properties of high organic C and N, and K content have led to its widespread reuse as a fertilizer supplement for sugarcane crops. Most often the vinasse is sprayed onto the fields, which is a process termed fertirrigation. This method is low cost and contributes to net energy savings in sugarcane bioethanol production cycles because the vinasse is locally transported and applied [4]. Benefits of using vinasse as fertilizer include improved short-term soil quality, crop production and crop quality [5–8]. However, negative effects include decreasing long-term soil fertility (lead leaching, N immobilization) and increasing greenhouse gas emissions, especially the emission of N_2_O when used in conjunction with an N fertilizer [2, 9–12]. These effects depend on the soil and environmental characteristics as well as vinasse-specific nutrient contents (reviewed in [12]). The increased N_2_O emissions from vinasse fertirrigation may be due to the stimulation of soil microbes by vinasse-derived organic material (i.e., a form of priming) and/or the activity of vinasse-derived cells containing genes in N_2_O-producing pathways [8].

Nitrous oxide emissions are produced through two main microbial-mediated processes in soil: nitrification (NH_4_^+^ to NH_2_OH to NO_3_^−^) and denitrification (NO_3_^−^ to NO_2_^−^ to NO to N_2_O to N_2_). Nitrification is carried out by microbes containing the ammonia monooxygenase enzyme, which is encoded by the gene *amo*A, and generally used as a functional marker of nitrifiers. Denitrifier bacteria may contain the nitrite reductase genes *nir*S and *nir*K, the nitric oxide reductase gene *nor*B and/or the nitrous oxide reductase gene *nor*B, which each encodes for the enzymes involved in the respiration of nitrite to nitric oxide to nitrous oxide to dinitrogen gas, respectively. The abundance of the different microbes containing denitrification genes, and the abundance of these genes when measured as functional markers, is known to correlate with the actual N_2_O emission rates from soils [62]. While much is known regarding the chemical characteristics of vinasse, there are only a few indirect studies of its biotic components despite recent attention to the environmental effects of its use in fertirrigation.

The microbiota present in vinasse likely encompasses the microorganisms present in the bioethanol production process. The most common raw material for ethanol production in Brazil is the mixture of diluted molasses and cane juice used in the distilleries annexed to sugar producing mills. The ethanol pipeline starts with crushing the unwashed sugarcane stalk to separate the sugarcane juice from the pulpy stalk residue known as bagasse. The sugarcane juice is heated and clarified with lime; the clarified juice is concentrated in an evaporator at 115 °C followed by vacuum boiling pan, at which point sugar and molasses crystallize. By centrifugation, the sugar crystals are separated from the mother liquor. This liquor is again crystallized in vacuum pans and then passed through continuous sugar centrifuges. The last residual solution is called molasses, which has high sucrose content suitable for ethanol production. The raw material for ethanol production is a mixture of unsterilized sugarcane juice, molasses and water [13]. The fermented material is then distilled at temperatures of at least 78 °C to separate the ethanol from the remaining waste vinasse. This vinasse is then transported via open channels or trucks to the sugarcane site for fertirrigation. The mixed sugarcane juice is fermented using proprietary *Saccharomyces cerevisiae* strains through two methods: batch (85% of distilleries as of 2011) or continuous fermentation (15%). In batch processing, the fermentation occurs in parallel, while in continuous fermentation the process occurs in series (reviewed in [14]). In either method, the yeast cells are treated with sulfuric acid, antibiotics, hop products and/or chemical biocides to reduce bacterial contamination, recovered by centrifugation, and reapplied to the fermentation tanks. This recycling step occurs between 400 and 600 times in a harvest season and despite the antibacterial treatment, bacteria remain the major contaminants.

The main bacterial contaminants of the bioethanol pipeline are lactic acid bacteria, which tend to dominate the samples taken from the ethanol pipeline in the steps prior to vinasse [15, 16]. These bacteria, in particular Lactobacillus species, compete with the commercial yeast strains for sugar or form exopolysaccharides that flocculate yeast cells [17–19]. Contamination by bacteria—through sucrose competition, flocculation of the commercial yeast strain or fermentation inhibition—can lower the efficiency of the bioethanol process by up to 30% [16, 20]. Furthermore, because of the antibiotic treatment of the yeast cells during the recycling step, contaminant bacteria may be a source of antibiotic resistance genes, as has been recently reported in a field study [21]. Other sources of contamination are wild yeast strains from the input sugarcane stalks, which are not sterilized prior to the production pipeline [22]. To date, no studies have investigated the presence of bioethanol pipeline contaminants in vinasse.

Here we investigated concurrently the chemical and microbial properties of vinasse to characterize the vinasse assemblage. We explored metagenomic data taken from vinasse samples over 1.5 years of production from a bioethanol mill in Piracicaba, SP, Brazil. The mill processes sugarcane produced in the region within a rough 40 km radius. Vinasse is distributed by trucks for fertirrigation during the harvest season (April to November). To characterize the microbial assemblage of this vinasse, we sequenced total DNA from six vinasse samples. We analyzed the resulting 18 shotgun metagenomes through metagenomics and differential abundance binning. To investigate the potential for N_2_O emissions from fertirrigation with vinasse, special attention was given to sequences and reconstructed genomes annotated as genes involved in N_2_O-related metabolism. Furthermore, we also identified genes relating to bioethanol production concerns to identify future research directions. To date, this is the first culture-independent study of the vinasse microbial assemblage. Our main questions were (1) what are the overall and sample-wise taxonomic and functional characteristics of the vinasse microbial assemblages? and (2) what is the potential of the vinasse microbes for N_2_O emissions, obstruction of fermentation and/or antibiotic resistance?

## Methods

### Sampling description

The bioethanol mill from which we sampled is in the region of Piracicaba in SP, Brazil. The mill takes in sugarcane from the region and produces sugar and ethanol. We obtained six time points of vinasse taken from transport trucks prior to their departure to the fields for chemical and molecular analyses. The trucks hold about 10,000 L of vinasse. Prior to sampling, the vinasse was held in the trucks for 24 h. Of the vinasse, 0.5 L sampled from the truck was immediately kept at 4 °C. The six sampling dates were 13/11/2013 (A, Nov. 2013), 13/12/2013 (B, Dec. 2013), 15/07/2014 (C, July 2014), 15/08/2014 (D, Aug. 2014), 14/10/2014 (E, Oct. 2014) and 10/11/2014 (F, Nov. 2014). The dates of the vinasse sampling corresponded to summer (October, November and December) or winter (July and August) sugarcane harvests. Because each vinasse was a random assemblage of contaminants from the bioethanol process, we considered each time point an independent measure for statistical analysis.

### Chemical analyses, DNA extraction, and qPCR quantification and sequencing

For each vinasse sample, 500 ml was used for chemical analyses. The remaining three subsamples of 100 ml per time point were used for DNA extraction. First, the samples were centrifuged at 10,621×*g* (Sigma 2-16P) for 10 min to separate cells from the liquid. Total DNA was extracted from the pellets with the MoBio PowerSoil kit according to the manufacturer’s instructions. Between 553 and 5310 ng was sent for sequencing (Additional file 1). The DNA was prepared as a MiSeq Illumina paired-end library and sequenced (3 replicates × 6 samples = 18 metagenomes) or used for quantitative PCR of genes that encode for the enzymes involved in the sequential biochemical steps leading to N_2_O production (*amo*A, *nir*K, *nir*S, *nor*B) or removal (*nos*Z). The qPCRs were performed in a 96-well plate (Bio-Rad) using CFX96 Touch™ Real-Time PCR Detection System (Bio-Rad). The qPCR reaction, primers combinations and thermal cycler conditions of each gene amplification are listed in Additional file 2. The qPCR data were acquired at 72 °C and melting curve analysis was performed to confirm specificity. Amplicon sizes were checked by agarose gel electrophoresis. Samples were analyzed with two technical replicates. Reaction efficiency varied from 80 to 105% and *R*^2^ values ranged from 0.94 to 0.99.

### Metagenome processing and read-based sample comparisons

Bioinformatics processing was performed on a Linux server (Linux-3.13.0-76-generic-×86_64-with-Ubuntu-14.04-trusty) with 64 nodes and 250 GB RAM. Processing was performed in a Snakemake v3.7.1 workflow or with bash or Perl scripts (available upon request). The 18 shotgun metagenomes were checked for tag sequences and evaluated for statistics using FastQC v0.10.1 (Available online at: http://www.bioinformatics.babraham.ac.uk/projects/fastqc) and PRINSEQ-lite version 0.20.4 [23]. Raw reads were filtered out using PRINSEQ if they had more than 1% of ambiguous (N) characters, had a mean quality score of less than 25 or were exact duplicates. Reads were trimmed at the 3′ end if the mean quality score was less than 20 within a sliding window size of 10 (clean reads). The clean paired-end reads were used in further analyses unless otherwise noted. The raw paired-end reads were merged using PEAR v0.9.5; these merged read ends were trimmed by quality and filtered out if the merged read had more than 1% ambiguous characters (parameters *q* 20, *n* 0.01) with PEAR (merged reads) [24]. For downstream normalization of annotation counts, calculations of average genome size per sample were carried out using MicrobeCensus [25]. To compare the metagenomes directly, sample distances were determined from the partial de Bruijn assembly of the clean forward reads using MetaFAST 0.1.0 (revision 57253a1) [26].

### Taxonomy, phylogeny and alpha diversity

To characterize the taxonomic composition, functional potential and diversity of the microbial assemblages in the vinasse samples, we profiled the metagenomes using different databases. First, the merged reads were uploaded to the metagenome analysis platform MG-RAST version 3.6 [27]. The metagenomes were compared using the default presets to the RefSeq or subsystem databases to obtain taxonomic or functional profiles, respectively. Refseq annotations, including eukaryota, bacteria, archaea and viruses, were determined using the last common ancestor approach. The MG-RAST taxonomic (phylum-level) and functional (Subsystems Level 1) profiles were analyzed with the statistical analysis of metagenome profiles (STAMP) software [28]. Taxonomic or functional level abundances significantly different among vinasse samples were evaluated using ANOVA. The Tukey–Kramer post hoc test with a 95% confidence interval and the Benjamini–Hochberg correction was used to identify differing phyla or Subsystems Level 1 category abundances between the vinasse metagenomes with significance determined at corrected *p* < 0.001 or 0.05, respectively. The taxonomic profiles at genus level were kept to visualize the relative abundance of genera across samples.

Because the metagenomes were well represented in the MG-RAST databases, we further characterized the taxonomy and functional potential of the metagenomes using metaphlan2 version 2.6.0 and humann2 version 0.9.9 pipelines [29, 30]. For metaphlan2 analysis, we used the “relab” analysis with the “–ignore_eukaryotes” flags to obtain taxonomic profiles. To gain an overall view of the taxonomy present in the vinasse samples and the phylogenetic relationships between the species in the samples, the average taxonomic distributions of the vinasse samples from metaphlan2 were visualized as a cladogram using Graphlan [31]. To examine the taxonomic profiles of vinasse across samples, these were visualized through heatmaps with average linkage clustering on Euclidean distances using hclust2. For the humann2 analysis, we annotated the forward clean reads against the UniRef90 database [32]. Pathway abundances were visualized excluding the “UNMAPPED” and “UNKNOWN” categories using hclust2 heat maps with average linkage clustering on Euclidean distances. To obtain a measure of alpha diversity, we ran metaphlan2 with previously mentioned flags on samples rarified to the smallest library size (280,161 reads).

To infer the phylogenetic relationships between the organisms present in the vinasse samples, full-length 16S rRNA genes were recruited from the vinasse metagenome reads using REAGO version 1.1 on forward clean reads truncated to 201 bp [33]. The resulting full-length 16S rRNA vinasse sequences were aligned and taxonomically classified against the SSU 128 SILVA reference database using SINA [34, 35]. The five nearest neighbors for each full-length 16S rRNA sequence were downloaded in addition to two *Verrucomicrobia* outgroup sequences. The 16S rRNA sequences were aligned without gaps using ClustalW in MEGA7 (121 sequences in total) [36]. A neighbor-joining tree was created with evolutionary distances computed using the Maximum Composite Likelihood method [37, 38]. Phylogenetic distances were evaluated with bootstrap tests (1000 replicates) [39]. To obtain a measure of alpha diversity we recruited full-length 16S rRNA genes using REAGO as above on the rarified metagenomes. Further, we evaluated a measure of genus-level relative abundance across samples by mapping the metagenome reads to the extracted 16S sequences grouped by taxonomic affiliation using bowtie2. These abundances were calculated as percentages of the number of aligned pairs from the total number of metagenome reads per sample.

### Putative denitrification and nitrification gene abundances

To investigate the potential for N_2_O emissions from the vinasse samples, we used two approaches: 1) metagenome read matching to profile HMMs of denitrification and nitrification genes and 2) recruitment of denitrifying and nitrifying genes from the reads. Profile HMMs for the *amo*A*_AOA, amo*A*_AOB, nir*K*, nir*S*, nor*B*, nosZ, nos*Z*_atypical_1* and *nos*Z*_atypical_2* genes were downloaded from the Functional Gene Repository (FUNgene version 8.3; available at http://fungene.cme.msu.edu/). Reads were translated to protein sequences with the “meta” setting using Prodigal version 2.6.2. The ORFs were queried for HMM matches using HMMsearch (command: hmmsearch –noali –o < filename.fasta > < gene.hmm > < filename.fasta > ; available at https://www.ebi.ac.uk/Tools/hmmer/search/hmmsearch). The HMM matches were normalized to reads per kilobase per genome equivalent (RPKG = (# mapped reads/HMM gene length (KB))/genome equivalents). The RPKG normalization accounts for genome size, library size and gene length biases, allowing for gene and sample comparisons.

In parallel, the gene-targeted assembler pipeline megagta version 0.1_alpha was used to recruit full-length genes from the metagenomes [40]. Gene-targeted assemblies (i.e., recruitments) were carried out on *amo*A_AOA, *amo*A_AOB, *nir*S, *nir*K, *nor*B_*cNor*, *nor*B_*qNor*, *nos*Z and *nos*Z_a2 genes using megagta. Further, to infer alpha diversity, the ribosomal *rpl*B gene was recruited from the rarified metagenomes.

### Cross-assembly and binning

We evaluated the performance of three assemblers (Ray-meta [41], Megahit [42] and metaSpades [43]) in cross-assembling the 18 vinasse metagenomes; the best cross-assembly was that from the metaSPADES assembler version 3.8.2 based on assembly characteristics evaluated using MetaQUAST (QUAST Version 3.0, build 07.07.2015 05:57 [44]). The 18 metagenomes were cross-assembled with metaSpades using kmer sizes 77, 99 and 127. The sample reads were mapped to the cross-contigs using bowtie2 to obtain cross-contig abundances [45]. The final metaSPADES cross-assembly was binned using three tools for comparison: CONCOCT (with anvio version 2.3.2), Metabat [46] and MaxBin2 version 2.1.1 [47]. The contig annotation tool (CAT version 2) was used to determine the taxonomic affiliation of all ORFs identified in each bin using prodigal to find ORFs and diamond blastp against the NCBI-nr database [48]. CAT taxonomy results were formatted using custom Perl scripts and visualized with TreeMap to aid with the taxonomic characterization of the bins. Because more genomes with > 90% completeness and coherent taxonomies were found from the MaxBin2 binning, these were selected for downstream analysis. CheckM was used to check the original MaxBin2 bins [49]. These bins were manually refined using anvi’o version 2.3.2 based on cross-contig taxonomy (from CAT), hierarchical clustering of the cross-contigs and sample coverage information [50]. The relative sample abundances of the bins were noted as the percent of sample reads recruited to the bin out of the total sample reads recruited to all the bins (i.e., percent recruitment anvi’o results).

The “good bins” were identified as having > 90% completeness and < 10% redundancy. Further “interesting bins” were further identified as those with > 20% completeness and < 10% redundancy and/or coherent contig taxonomies. Functional annotation of the “good and interesting bins” were carried out using prokka with the “kingdom” flag set to bacteria or viruses depending on the taxonomic classification [51]. To characterize the bins by their potential functional type, prokka annotation results were mined for lines matching EC numbers of KEGG enzymes of compounds related to bioethanol production interests and N_2_O emissions. These KEGG compounds were acetate (C00033), cellulose (C00760), xylose (C00181), lactose (C00242), caproic acid (C01585, carbon dioxide (C00011), diacetyl (C00741), hydrogen peroxide (CC00027), lactaldehyde (C05999) and phenyl lactate (C05607). The lists of EC numbers were obtained by querying the KEGG REST API on each compound ID. Keyword searches of “3-hydroxy” fatty acids, “cyclic dipeptide”, antibiotic “resistance”, and nitrification and denitrification genes were additionally used to identify the potential presence of these functions in the bins.

In parallel, to confirm potential denitrification and nitrification gene presence, bin sequences were compared to HMMs of nitrification and denitrification genes from FunGene as described previously but with the prodigal setting “single.” The HMM matches were normalized by bin size (number of ORFs and total number of bp in ORFs) and HMM length in bp.

## Results

### Vinasse chemical characteristics and metagenome overview

The chemical characteristics of the vinasse samples are listed in Table 1. Average pH was low at 4.4 ± 0.4, ranging between 3.9 (D) and 4.8 (C). Total organic carbon averaged 29 ± 1.8 g/L and ranged between 25.7 (B) and 31.4 g/L (D). Total N averaged 0.64 ± 0.15 g/L, while that of P and K was 0.16 ± 0.07 and 3.43 ± 1.02, respectively. The C/N ratio averaged 42 ± 13 and ranged between 19 (F) and 57 (C). After processing, the 18 vinasse metagenomes contained a total of 2,126 Mbp distributed into 7.82 million reads. The number of reads ranged between 280,161 and 542,208 sequences per sample with between 77 and 150 Mbp (Additional file 1). When the metagenome distances were compared using partial de Bruijn assembly, A and C were most similar, followed by F, followed by E; least similar were B and last D (Additional file 3).

**Table 1:** Chemical characteristics of the six vinasse samples

Group name	Sampling date	pH	C org (g/L)	N tot (g/L)	N-NH_4_^+^ (mg/L)	N-NO_3_^−^ (mg/L)	P (g/kg)	K (g/kg)	C:N
A	Nov. 2013	4.7	28.2	0.53	65.8	17.6	0.08	2.9	53
B	Dec. 2013	4.1	25.7	0.53	63.4	10.8	0.17	2.6	49
C	July 2014	4.8	28.8	0.51	45.7	8.8	0.11	3.5	57
D	Aug. 2014	3.9	31.4	0.89	41.6	4.1	0.23	4.7	35
E	Oct. 2014	4.2	29.6	0.74	37.7	6.8	0.10	2.1	40
F	Nov. 2014	4.7	30.3	1.57	75.9	6.6	0.25	4.8	19

### Taxonomic characterization

**Fig. 1:**
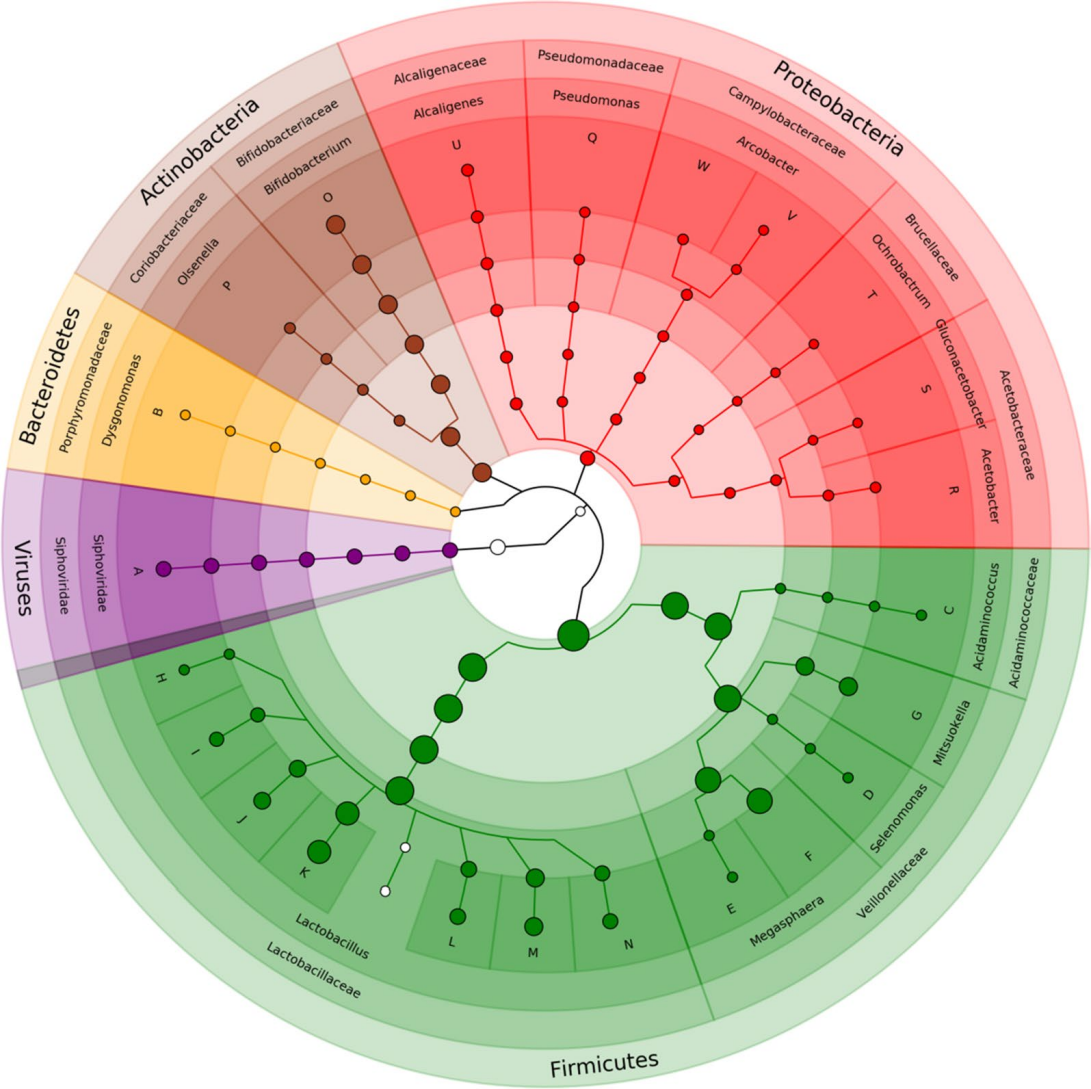
Average abundance of taxa in the vinasse samples. The metagenomes were analyzed using metaphlan2 and visualized with GraPhlan. Node sizes correspond to average relative abundance across the vinasse metagenomes while colors correspond to phylum. Species are noted with letters: A = *Lactobacillus* phage Lc Nu, B = *D. mossii*, C = *A. intestini*, D = *S. bovis*, E = M. elsdenii, F = Megasphera unclassified, G = Mitsuokella unclassified, H = L. salivarius, I = L. equicursoris, J = L. delbrueckii, K = L. amylovorus, L = L. mucosae, M = L. fermentum, N = L. vini, O = B. thermophilum, P = Olsenella unclassified, Q = Pseudomonas unclassified, R = Acetobacter unclassified, S = Gluconacetobacter unclassified, T = Ochrobactrum unclassified, U = A. faecalis, V = A. butzleri and W = Arcobacter unclassified

**Fig. 2:**
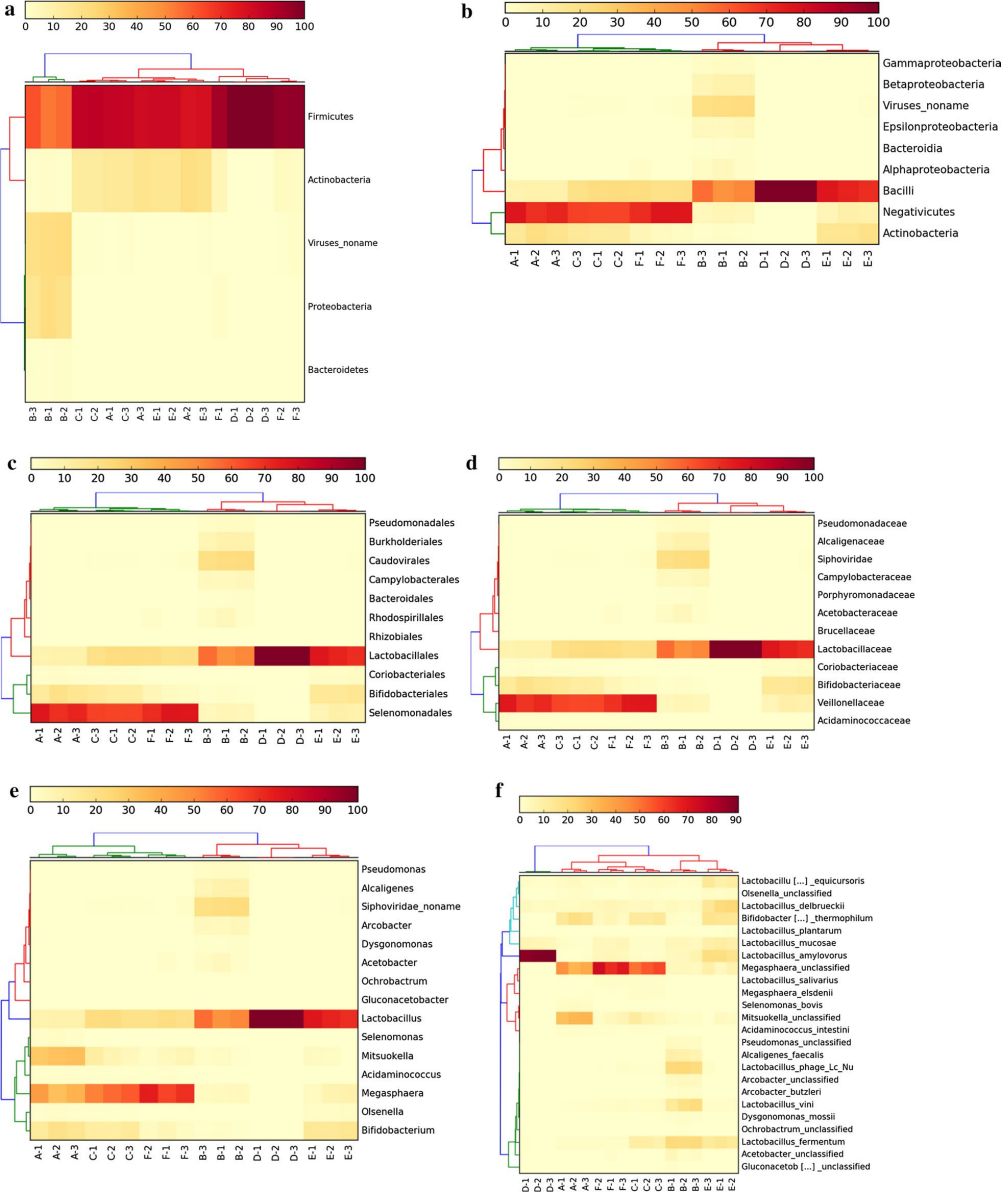
Taxonomic distributions across the vinasse samples at the level of** a** Phylum,** b** Class,** c** Order,** d** Family,** e** Genus and** f** Species. The taxonomic group and sample profiles were clustered using hclust2 from metaphlan2 results

When compared to the M5NR database containing eukaryota, bacteria, archaea and viruses on MG-RAST (Additional file 4), 21–55% of the merged reads could be classified. Of the classified reads, 96–100% were annotated as bacteria. The top phyla present in the vinasse samples with relative abundances greater than 1% and/or that significantly co-varied among the samples (ANOVA at *p* < 0.001 and Kruskal–Wallis post hoc test) were Firmicutes (35–97% of merged reads), Bacteroidetes (0.8–53%), Actinobacteria (0.4–17.5%) and Proteobacteria (0.3–39.4%; Additional file 5). The “core” phylum observed in all vinasse samples was Firmicutes. Similarly, when compared to the metaphlan2 marker gene database containing bacteria, archaea and viruses (excluding eukaryotes), between 68 and 100% of classified reads were identified as bacteria and 0–32% as viruses (Fig. 1). The previous four main bacterial phyla again dominated the vinasse samples: Firmicutes (48–100% of classified reads), Actinobacteria (0–19%) and Proteobacteria (0–18%), as well as viruses (0–32%; Fig. 2). The most abundant bacterial genera were Lactobacillus (Phylum Firmicutes), Megasphaera (Firmicutes), Mitsuokella (Firmicutes) and Bifidobacterium (Actinobacteria). Further supporting these taxonomic results, the full-length 16S rRNA genes recruited from the vinasse metagenomes were classified as Bifidobacterium (Phylum Actinobacteria), Olsenella (Phylum Actinobacteria), Prevotella (Phylum Bacteroidetes), Lactobacillus (Phylum Firmicutes), Megasphaera (Phylum Firmicutes), Mitsuokella (Phylum Firmicutes) and *Comamonas* (Phylum Proteobacteria) genera (Additional files 6, 7).

When the samples were clustered based on the MG-RAST taxonomic profiles at phylum level, E and C formed a cluster while A, F, and D were separated based on the first principal component and B was separated based on the second (Additional file 8). When the metaphlan2 profiles were clustered at the level of class, order, family and genus, samples A, C and F formed a cluster while B, D and E formed a separate cluster (Fig. 2).

### Functional potential characterization

**Fig. 3:**
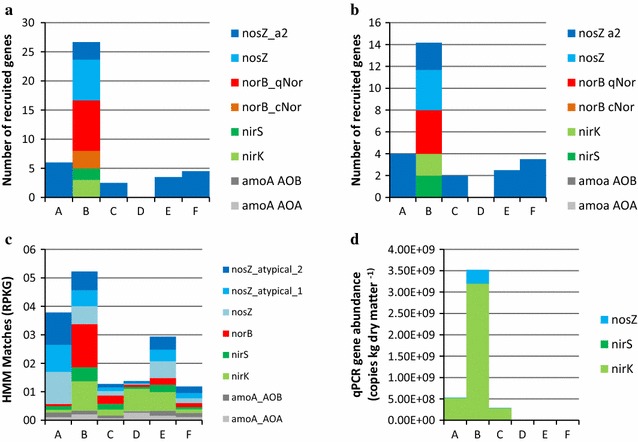
Putative gene abundances in the vinasse metagenomes. Partial gene fragments were recruited from the vinasse metagenomes using megagta on (**a**) all reads and (**b**) rarified reads. In parallel, vinasse metagenomes were compared to profile HMMs and the number of matches was normalized to (**c**) reads per kilobase per genome equivalent (RPKG). In (**d**) the gene copy numbers from real-time PCR of the nosZ, nirS and nirK genes are depicted. Note that no qPCR of the norB gene was made

When compared to the M5NR databases through MG-RAST, the percentage of sequences with ORFs that could be classified into functional categories ranged between 16 and 42% (Additional file 9). At Subsystems Level 1, the top significantly different categories were carbohydrate metabolism, clustering-based subsystems, amino acids and derivatives, miscellaneous, protein metabolism, DNA metabolism, RNA metabolism, cofactors/vitamins, cell wall/capsule, phages/prophages and nucleosides and nucleotides. When sample distances were determined using the functional profiles at Subsystems Level 1, C, A, B and F formed a cluster while E was separated based on the first principal component and D was separated based on the second (Additional file 10). When the vinasse metagenomes were analyzed using the humann2 framework, abundant pathways were found in sample D, which was dominated by one *Lactobacillus*—the top abundant pathways included PWY-5100: pyruvate fermentation to acetate and lactate II and PWY-7219: adenosine ribonucleotides de novo biosynthesis (Additional file 11). Combining the real-time PCR, gene recruitment and gene mapping results, the vinasse metagenomes had few to no genes matching nitrification genes; in contrast, a range of denitrification genes was found (Fig. 3). Sample B presented the most diversity of denitrification genes, with *nir*K, *nir*S, *nor*B and *nos*Z present based on the recruitment and mapped results. The presence of putative *nos*Z was supported in all samples except D. In addition, putative *nir*K was found in all samples except F.

### Alpha diversity of the vinasse samples

Several methods were employed to obtain estimates of the alpha diversity of the vinasse samples (Table 2). The normalized effective number of species from MG-RAST averaged 29 ± 14 and ranged between 3 (D) and 53 (B) species. When metaphlan2 was applied to the rarified vinasse samples, the number of classified species averaged 11 ± 3 and ranged between 5 (D) and 14 (B) species. Partial 16S rRNA fragments recruited from the rarified samples using REAGO averaged 10 ± 4 and ranged between 4 (D) and 17 (E). Further, when the *rpl*B gene was recruited from the rarified samples with megaGTA analysis, and average of 17 ± 3 fragments could be found across the vinasse samples with between 13 (E) and 22 (C) *rpl*B fragments identified. When the 16S rRNA gene was amplified using real-time PCR of the vinasse samples, the number of genes per kg of dry matter averaged 12e12 ± 9e12 and ranged between 0.8 (E) and 25.7 (A); the gene abundance of 18S rRNA gene averaged 100e3 ± 71e3 and ranged between 17e3 (D) and 208e3 (B).

**Table 2:** Alpha diversity estimates of the vinasse samples

Sample name	REAGO	megaGTA	metaphlan2	MG-RAST	qPCR
	Number of Recruited 16S rRNA genes	Number of Recruited rplB genes	Number of Species	Number of Effective species	Number of 16S rRNA copies (/1,000,000) kg dry/matter
A	13 ± 2	21 ± 2	10 ± 1	37 ± 1	25,750 ± 13,900
B	10 ± 3	16 ± 3	14 ± 1	47 ± 4	16,839 ± 11,664
C	12 ± 1	22 ± 2	13 ± 0	38 ± 1	16,281 ± 1104
D	4 ± 0	15 ± 2	5 ± 0	3 ± 0	10,749 ± 3336
E	17 ± 2	13 ± 1	10 ± 0	20 ± 1	839 ± 840
F	6 ± 2	17 ± 1	12 ± 1	29 ± 3	1135 ± 1142

Diversity was quantified by the number of partial genes recruited (REAGO and megaGTA), or the estimated number of species (metaphlan2 and MG-RAST) from the vinasse metagenomes; results from real-time PCR of the 16S gene were also included. Rarified forward reads were used as input for metaphlan2, reago and megagta analysis; merged reads were used in the MGRAST analysis and these results were normalized by library size

### Bin characteristics, taxonomy and functional types

The cross-assembly resulted in 221,975 cross-contigs totaling 216 Mbp. Of the cross-contigs, 40,815 were longer than 1Kbp, and 40,186 of these could be binned. After refining the bins, 20,825 cross-contigs remained distributed within the 36 good or interesting large bins (0.6–3.9 Mbp; hereafter referred to as the large bins). The large bins represented 39–68% of the sample reads. Fifty-eight percent of the large bins were classified at the phylum level as Firmicutes, 8% as Bacteroidetes, 17% as Proteobacteria, 11% as Unknown and 6% as Actinobacteria (Table 3). Overall, the GC percent of these bins ranged between 28 and 66%. Of the large bins, 24 were potential denitrifiers and three potential nitrifiers. The presence of genes related to acetate, CO_2_, ethanol, H_2_O_2_ and lactose metabolism was found in all large bins while the potential presence of genes related to lactaldehyde, mannitol, xylose, butyric acid, cellulose, diacetyl, phenyl lactate, sucrose and “3-hydroxy” was variable across the large bins (Table 4). Last, when multidrug resistance was identified in the bin annotations, all large bins but Unknown-19 and Lactobacillus-30 contained these genes. In addition to the large bins, eight small bins (0.03–0.20 Mbp) lacking bacterial marker gene presence were found (Table 5 and Additional files 12 and 13). The largest of the small bins, 4.2 and 8.1, were most abundant in samples E and D, respectively.

**Table 3:**
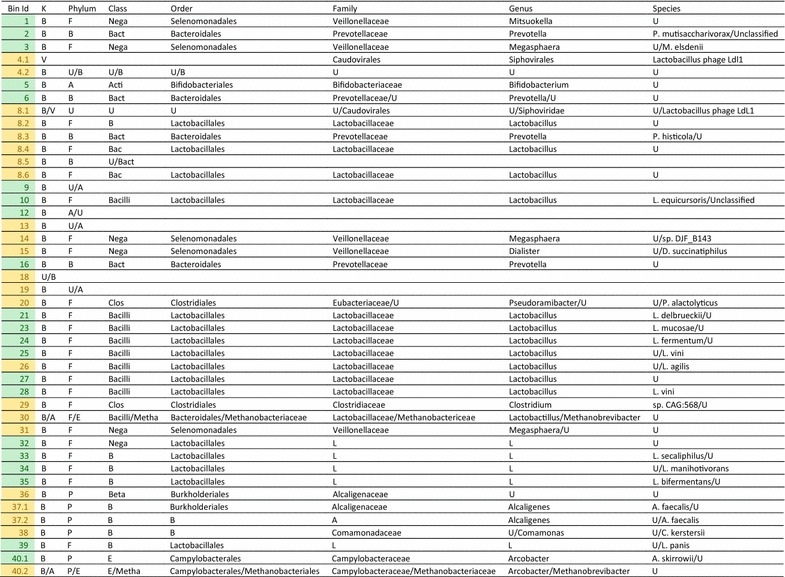
Taxonomy of the “good and interesting” vinasse bins based on CAT classification

K, Kingdom; F, *Firmicutes*; B, *Bacteroidetes*; A, *Actinobacteria*; P, *Proteobacteria*; E, *Euryarchaeota*; U, Unknown; Bact, *Bacteroidia*; Nega, *Negativicutes*; Clos, *Clostridia*; Mega, *Megasphaera*; Lact, *Lactobacillales*; Metha, *Methanobacteria*

**Table 4:**
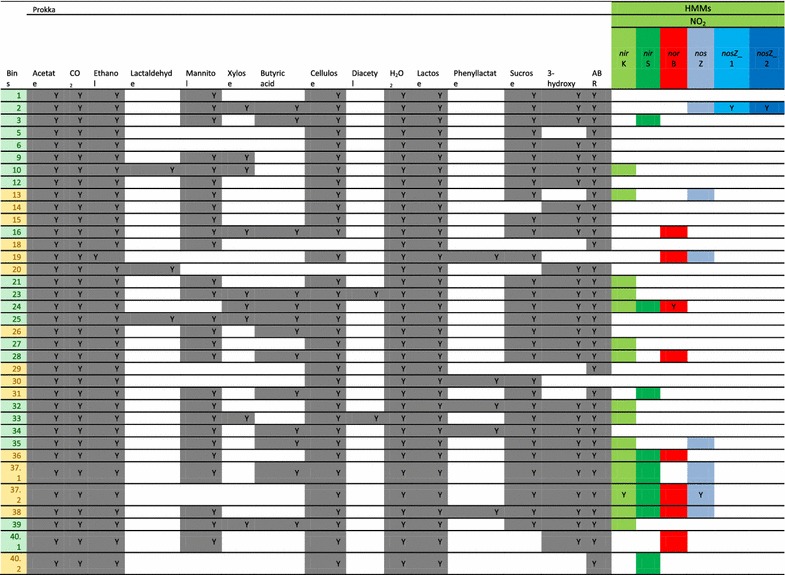
Putative gene repertoires of the large vinasse bins

Keyword searches of prokka annotation results (gray, “Y”) were supplemented in the case of the N_2_O metabolism-related genes with hmm profile search results (colors). Substrates for the genes related to N_2_O metabolism are included (colors above genes)

No genes related to the metabolism of caproic acid were found in the bin annotations. No *amo*ABC, *hao*, *nxr* *nor*, *nirS* genes were found in the bin annotations, but the *amo*A AOA gene was identified in Bin 23 and 40.1 and the *amo*A AOB gene was identified in Bin 33 by HMM matches

*ABR* antibiotic resistance

**Table 5:**
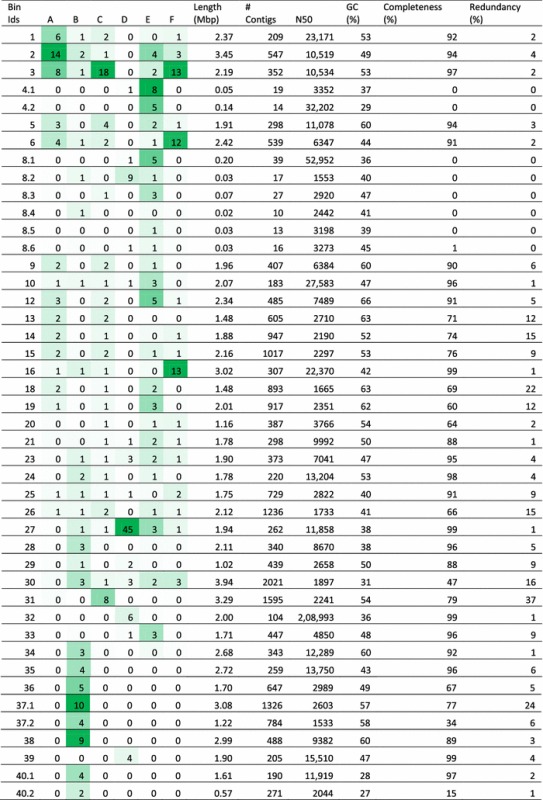
The “good and interesting” vinasse bin characteristics and relative sample abundances (indicated by heatmap per sample

## Discussion

Here, we explored concurrently the chemical and microbiological characteristics of vinasse produced over 1.5 years from one bioethanol mill in Sao Paulo State. The aims were to characterize, for the first time, the taxonomy and potential functions of the microbial assemblage in vinasse; we further recovered draft genomes from vinasse bacteria. We combined metagenomic analyses with binning techniques to characterize the vinasse assemblages and bacteria, respectively. We discuss below both potential ethanol pipeline contamination traits of vinasse bacteria and the potential ecology of vinasse fertirrigation. The vinasse chemical characteristics fell within the range of other sugarcane vinasses [52, 53]. Different vinasse inputs are known to contribute different nutrition; this is taken into account in that vinasse fertirrigation is applied depending on the amount of K present in the input vinasse [54]; however, that different vinasse inputs contribute different bacteria was not known until now. The different nutrient contents of vinasse originate from the differences in the input of sugarcane stalks to the bioethanol production process; this might also be the source of the vinasse bacteria.

The vinasse draft genomes most likely represented the bacteria that survived the selective bottleneck of the bioethanol production pipeline. The potential for bacteria found in vinasse originating from later steps in the bioethanol pipeline, such as the truck from which we sampled the vinasse, was considered a minor source of bacteria due to the large capacity (10,000 L), making this a negligible source of bacteria. The core genus found in the vinasse samples was *Lactobacillus* (Phylum Firmicutes), which is a previously known ubiquitous ethanol pipeline contaminant due to its tolerance of low pH [15]. Other known contaminants found prior to the distillation stage that we observed in our vinasse samples included representatives of the *Acetobacter*, *Bacillus*, *Bifidobacterium*, *Clostridium*, *Gluconacetobacter*, *Lactobacillus* and *Pseudomonas* genera [16, 55, 56]. Strikingly, we identified members of the genera *Megasphaera* and *Mitsuokella* that have not previously been reported as bioethanol pipeline contaminants. Members of the genus *Megasphaera* and *Mitsuokella* are Gram-negative ruminant fermenters that have been found in pig hindguts, cow rumen and human dental plaque and feces; Gram-positive *Bifidobacterium* have also been used as probiotics in humans and are found in the gut, vagina and mouth of mammals and bovine rumens. Whether these bacteria interact with each other within each vinasse sample—e.g., *Megasphaera* and *Mitsuokella* utilizing lactose provided by *Lactobacillus*—is unknown, as is the direction of the interactions.

Uncovering the physiological mechanisms by which these particular bacteria survive the selection bottlenecks of the bioethanol process was outside the scope of the current research since our goals were to characterize fully the metagenomic data. However, we speculated that plausible protective mechanisms are biofilm formation [16, 57], strain-dependent temperature tolerance, and unknown pipeline management considerations. For the latter, the distillation material might not homogenized, thus creating pockets of lower temperatures where the bacteria can remain. Other management considerations that might affect the viability of bacterial cells are length of time exposed to the distillation temperature and the highest temperature reached. Evaluating the physiology of cultured isolates from vinasse, which can be done building upon the work described here, is an interesting topic for further research.

Here, using differential abundance binning, we successfully obtained 21 draft genomes from vinasse bacteria likely representing bioethanol contaminants. We confirmed that roughly half of the vinasse bins were of the genus *Lactobacillus* (Phylum Firmicutes), which is the most ubiquitous bacterial bioethanol pipeline contaminant [16]. We also uncovered contaminants with up to 70% of sample coverage from the *Prevotella* (Phylum Bacteroidetes), *Megasphaera* (Phylum Firmicutes), and *Mitsuokella* (Phylum Firmicutes) genera, which have not been well studied. Five of the draft genomes were from bacteria unknown at the phylum level. Furthermore, most of the bins recovered here were partly uncharacterized at the species level, supporting the idea that we obtained genomes from novel strains of bioethanol contaminants. Studies of bioethanol contaminants to date have used culture-based methods, which do not capture the entire microbial diversity, or profiling of 16S rRNA genes, which does not capture the functional potential of the contaminants  [15, 56]. Bacterial contaminants in general are known to compete with commercial yeast strains, lowering ethanol yield; contaminants may also flocculate with the yeast or produce compounds such as acetate, butyric acid or lactose which might inhibit yeast fermentation [16]. Many bins contained sucrose metabolism-related genes, suggesting that these might compete with the commercial yeast strain for sugarcane sucrose. Annotation of the bins revealed the potential presence of bioethanol contaminant genes related to the metabolism of acetate, ethanol, mannitol, cellulose, hydrogen peroxide, lactose, sucrose and 3-hydroxy fatty acids. These results support the idea that vinasse bacteria are an additional source in identifying likely bioethanol process contaminants.

Interesting bins included *Lactobacillus/Methanobrevibacter*-bin30 and Arcobacter/Methanobrevibacter-bin40.2, which contained cross-contigs annotated as both bacterial and archaeal. *Methanobrevibacter* is an archaeal genus whose methanogenic members are often found in vertebrate guts consuming the end products of bacterial fermentation. Finding them here suggests that this interaction might also be present in vinasse. In addition, we binned potential phage genomes, which suggest that phages are present in the fermentation tanks along with the host contaminants. The large phage genome bin 8.2 was most abundant in vinasse sample D, corresponding to a low-diversity assemblage with a dominant bin, suggesting that the host of this phage was *L. amylovorus*-bin27. The phage bins 4.1, 4.2 and 8.1 were all most abundant in vinasse sample D, corresponding to a more diverse assemblage of bacterial hosts across the phyla Firmicutes and Bacteroidetes. These associations suggest that phage lysis may be a factor controlling bacterial population sizes in the fermentation tanks. Attention has recently been paid to using phage therapy to control bacterial contamination in bioethanol pipelines [58, 59].

In addition to investigating the potential for vinasse bacteria to be contaminants in the production of bioethanol, we evaluated the potential for vinasse bacteria to contribute to N_2_O emissions during fertirrigation. Vinasse fertirrigation can be considered a disturbance on the soil microbial community; the success of the vinasse assemblage in the soil likely depends on the connectivity (e.g., strength and direction of the vinasse species interactions). Pitombo et al.  [11] identified significantly abundant bacterial genera under treatments of vinasse compared to control plots without vinasse using 16S rRNA gene marker abundance, and the significantly differentially abundant genera in the plots amended with vinasse included the vinasse bacteria (as identified here) *Lactobacillus*, *Bacillus*, *Prevotella*, *Gluconacetobacter*, *Megasphaera*, *Mitsuokella* and *Acetobacter* [11]. Further, unpublished research suggests that vinasse bacteria on a field experiment may persist at low abundances. These results together suggest that vinasse bacteria may successfully invade the soil microbial community [60]. Furthermore, the vinasse bacteria may transfer to the sugarcane stalks during plant growth and at harvest time become the contaminants that are inputted with the sugarcane to the ethanol processing pipeline. In support, a survey of the bacteria associated with the sugarcane plant found the vinasse taxa *Bacillus*, *Acetobacter* and *Gluconacetobacter* as part of the “core” sugarcane microbiome [22]. While this is an interesting speculation, we note that caution should be taken because the referenced studies were few and based on gene marker surveys at higher taxonomic levels, which hinders robust and precise interpretation. We recommend further research into the ecological interactions of vinasse bacteria with the soil bacterial community at the species or strain level during fertirrigation with vinasse.

Actual N_2_O emissions from a soil are the result of the sequential biochemical processes, nitrification and denitrification, carried out collectively by the microbial communities in a soil. The total rate of N_2_O emissions through nitrification or denitrification is controlled by carbon availability, moisture, oxygen availability, pH, temperature, and nitrate concentrations. These factors limit enzyme activity, gene transcription levels and microbial cell growth [61]. Furthermore, the abundance of the genes involved in the production (*amo*A, *nir*K, *nir*S, *nor*B) or removal (*nos*Z) of N_2_O is correlated with the actual N_2_O emissions [62]. In the case of vinasse fertirrigation, vinasse contributes with microorganisms associated with N_2_O emissions but they are not the sole source and probably not the most important related to N_2_O emissions. Do Carmo et al. [10] and Pitombo et al. [11] have shown that at early stages, soon after vinasse application together with inorganic N fertilizer there is an increase of N_2_O emissions [10, 11], we hypothesize that this is because of a combination of factors present in vinasse (organic C, organic N and microbes with genetic repertoires for denitrification) that will interact with soil microbiome and soil environmental conditions. Disentangling the interaction of each biotic and abiotic factor present in vinasse with soil existent microbes related to N_2_O emission and soil environmental factors is an interesting topic for future research.

Four phyla (Firmicutes, Actinobacteria, Proteobacteria and Bacteroidetes) were represented across the vinasse samples, although at the genus level the diversity of each assemblage fluctuated. The samples could generally be classified as dominated by *Megasphaera* (A, C, F) or *Lactobacillus* (B, D, E) at the genus level. The second assemblage (B) was the most diverse; it was dominated by *Lactobacillus* and containing, uniquely compared to the other time points, Proteobacteria such as *Alcaligenes*, as well as phage (*Lactobacillus* phage Lc Nu). The least diverse assemblage was D, containing mostly *Lactobacillus* and phage. Of the 22 potential vinasse denitrifiers, two were potential complete denitrifiers (containing *nir*K or *nir*S, *nor*B and *nos*Z) and eight were potential incomplete denitrifiers (containing *nir*K or *nir*S and *nor*B). The abundances of these potential denitrifiers varied across time points, suggesting varied effects on N_2_O during vinasse fertirrigation with different vinasses. For example, the *Lactobacillus*-bin 27 dominated to 97% of the sample D abundance, and this contained a putative *nir*K gene; when this vinasse is sprayed onto the fields, one would expect nitrate degradation and an increase in N_2_O or N_2_ depending on the gene content of the endogenous microbial community. Another abundant potential denitrifier present in sample A (*Prevotella*-Bin 2) contained only potential *nos*Z, suggesting that if the vinasse A was to be used in fertirrigation, the actual emission of N_2_O may be reduced due to the further reduction of N_2_O into N_2_. Furthermore, vinasse denitrifiers might directly contribute to the N_2_O emissions observed when vinasse is added in conjunction with a nitrate fertilizer. This suggests that vinasse application in conjunction with a reduced nitrogen source such as ammonium sulfate may be a feasible management practice to reduce N_2_O production. Further research investigating the microbes involved in N_2_O emissions during fertirrigation with vinasse would greatly aid in steering future vinasse management strategies.

Vinasse fertirrigation has raised human health concerns that vinasse bacteria may carry antibiotic resistance genes (ARGs) [21]. These genes can enter the soil resistome and can be transferred using horizontal gene transfer to other soil bacteria, with potential spreading of antibiotic resistance genes to soil-derived human pathogens. Here a search of the annotation results of the recovered vinasse bins found multidrug resistance genes in 34 of the 36 large bins. Surprisingly, no drug resistance genes were found in the phage bins; this may indicate that the phages from which these genomes were not prophage that confer auxiliary metabolic genes in the form of antibiotic resistance to the vinasse bacteria. These results warrant further study of the fate of ARGs from vinasse during fertirrigation.

While significant progress has been made in metagenome assembly and binning, some caveats should be noted to the bins we recovered here. Misassembly and misbinning can occur and bias the final results, in our case identifying relevant genes present in the bins. We addressed these issues by comparing three assemblers and three binning tools and choosing the best of each. Further, we used large kmer sizes for the final cross-assembly, and this successfully allowed MaxBin2 to bin to the level of species. We additionally used the manual refinement feature of anvi’o to improve the bins. Because bins with low completeness as determined by the presence of universal marker genes can still contain useful information regarding potential gene content, we used all useful bins to characterize the vinasse assemblage. Eight bins could not be refined and these represent unbinned vinasse bacterial genome content; however, the information from this genomic material was characterized in the metagenomic analyses. We included several different methods for each analysis to supplement each other as database coverage and read length can bias results based on sequence alignment. Moreover, we used the metagenomic analysis to complement the bin results. Interestingly, comparing the qPCR, putative gene abundances and gene recruitment results suggested that the qPCR primers we used do not cover the entire diversity of vinasse bacteria or alternately that the HMM results may be biased toward false positives.

Here we used metagenomic analysis and genome binning to characterize in depth the assemblage of six vinasse samples from one bioethanol mill. We identified previously unknown vinasse taxa compared to taxa identified through culture-or 16S rRNA survey-based studies of the ethanol processing pipeline steps prior to vinasse. Furthermore, we obtained 21 draft bacterial genomes and 8 draft phage or mobile element genomes from vinasse, which to our knowledge is the first study to do so. Vinasse bacteria included mainly putative denitrifiers, which may directly affect soil N_2_O or N_2_ emissions when applied during fertirrigation, although more research is needed into the ecological interactions during this event. In the vinasse bins we found the putative presence of antibiotic resistance genes and genes affecting yeast fermentation, which potentially implicate vinasse bacteria in negative impacts on human health and bioethanol production, respectively. We suggest that monitoring the vinasse assemblage is a promising option to screen both for bioethanol production contaminants and to identify vinasse batches which, when added to the fields during fertirrigation, may lead to higher N_2_O emissions. Because of the decreasing costs of high-throughput sequencing, we suggest that monitoring of vinasse assemblages can be widely implemented to improve sugarcane bioethanol production sustainability.

## Additional files

**Additional file 1.** Data description of the 18 vinasse metagenomes.

**Additional file 2.** Primers and thermocycler conditions used in gene abundance analysis by real time qPCR of the vinasse samples.

**Additional file 3.** Hierarchical clustering of the vinasse metagenomes based on partial de Bruijn graph overlap from Metafast analysis. Replicates were most similar to each other.

**Additional file 4.** Data description of the merged vinasse metagenomes uploaded to MG-RAST.

**Additional file 5.** Taxonomic distribution of the merged vinasse metagenomes from MG-RAST annotation against RefSeq database. Phyla with average relative abundance greater than 1% across all samples were included. Samples with significantly different phyla between groups (Tukey–Kramer post hoc test, 95% confidence interval, *p* < 0.001) are indicated by different letters.

**Additional file 6.** Phylogenetic relationships between full-length 16S rRNA genes reconstructed from the vinasse metagenomes using REAGO. The 16S rRNA sequences from vinasse and reference sequences were aligned using ClustalW. The neighbor-joining tree was created with MEGA and visualized using iTol ignoring branch lengths.

**Additional file 7.** Functional potential characterization of the vinasse samples from MG-RAST annotation against the Subsystems database. Only the subsystems at level 1 with average relative abundance greater than 2% across all samples were included. Significantly different subsystems at level 1 between sample groups (Tukey–Kramer post hoc test, 95% confidence interval, *p* < 0.05) are indicated by different letters.

**Additional file 8.** Principal component analysis of the Subsystems Level 1 category abundances of the vinasse samples. The colors correspond to time point. Profiles were determined against the Subsystems database using MG-RAST and relative abundances of phyla were calculated out of the total number of sample reads.

**Additional file 9.** Functional potential profiles of the top 30 pathways across the vinasse samples, excluding “unmapped” and “uncategorized” results. The functional group and sample profiles were clustered using hclust2 from humann2 analysis against the UniRef90 database.

**Additional file 10.** All vinasse bin characteristics and relative sample abundances (indicated by heatmap per sample). Bin id’s highlighted in green indicate “good” bins; yellow id’s indicate “interesting” bins, and red indicate “bad” bins.

**Additional file 11.** All vinasse bin taxonomic affiliations based on CAT classification. Bin id’s highlighted in green indicate “good” bins; yellow id’s indicate “interesting” bins, and red indicate “bad” bins.

**Additional file 12.** All vinasse bin taxonomic affiliations based on CAT. Bin id’s highlighted in green indicate “good” bins; yellow id’s indicate “interesting” bins, and red indicate “bad” bins.

**Additional file 13.** Principal component analysis of the phylum-level abundance distributions of the vinasse samples. Relative abundance profiles were determined using MG-RAST against the RefSeq database and phyla membership was determined using the Last Common Ancestor algorithm.

## Abbreviations

MG-RAST: metagenomics analysis server
*nir*K, *nir*S: nitrite reductase genes
*nor*B: nitric oxide reductase gene
*nos*Z: nitrous oxide reductase gene
*amo*A: ammonium monooxygenase gene
ABR: antibiotic resistance gene
