# Correction to: Genome-resolved metagenomics of sugarcane vinasse bacteria

**DOI:** 10.1186/s13068-018-1254-1

**Published:** 2018-10-03

**Authors:** Noriko A. Cassman, Késia S. Lourenço, Janaína B. do Carmo, Heitor Cantarella, Eiko E. Kuramae

**Affiliations:** 1 Department of Microbial Ecology, Netherlands Institute of Ecology NIOO-KNAW, Wageningen, Netherlands; 2 Soils and Environmental Resources Center, Agronomic Institute of Campinas, P.O. Box 28, Campinas, SP 13012‑970 Brazil; 3 Environmental Science Department, Federal University of São Carlos, Sorocaba, SP 18052‑780 Brazil

## Correction to: Biotechnol Biofuels (2018) 11:48 10.1186/s13068-018-1036-9

The original version of the article contained a mistake. The accession number has been incorrectly published in the “Availability of data and materials” section.

The number is given below and has been corrected with this erratum.

The datasets generated and analysed during the current study are available in the MG-RAST repository with identification numbers described in Additional file 4.

The NCBI shotgun metagenomes are BioProject id: PRJNA435511. Draft bacterial genomes are available at https://zenodo.org/record/1194340 (DOI: 10.5281/zenodo.1194340).
