# Supplementary material for: Genome-resolved metagenomics of sugarcane vinasse bacteria
Source: Biotechnol Biofuels. 2018 Feb 22;11:48. doi: 10.1186/s13068-018-1036-9 (PMC5822648; doi:10.1186/s13068-018-1036-9)
Supplement: Supplementary file 1 — Additional file 1. Data description of the 18 vinasse metagenomes. [file 13068_2018_1036_MOESM1_ESM.docx]

**Genome-resolved metagenomics of sugarcane vinasse bacteria**

Noriko A. Cassman^1^, Késia S. Lourenço^1,2^, Janaína B. do Carmo^3^, Heitor Cantarella^2^, Eiko E. Kuramae^1^

^1^Department of Microbial Ecology, Netherlands Institute of Ecology NIOO-KNAW, Wageningen, Netherlands

^2^Soils and Environmental Resources Center, Agronomic Institute of Campinas, P.O. Box 28, 13012-970, Campinas, SP, Brazil

^3^Environmental Science Department*,* Federal University of São Carlos, 18052-780, Sorocaba, SP, Brazil

Correspondence: EE Kuramae, Department of Microbial Ecology, Netherlands Institute of Ecology NIOO-KNAW, Wageningen, Netherlands. Email: [e.kuramae@nioo.knaw.nl](mailto:e.kuramae@nioo.knaw.nl)

**Additional file 1.** Data description of the 18 vinasse metagenomes.

| Date  Sampled | Sample  Name | Sample Id | DNA Weight  (g) | DNA Conc.  (ng/µl) | # reads | Forward  # bases (Mbp) | Reverse  # bases (Mbp) | Reads mapped to  cross-contigs (%) |
| --- | --- | --- | --- | --- | --- | --- | --- | --- |
| Nov.  2013 | A-1 | 1V1-1 | 0.297 | 32.5 | 461,784 | 131 | 117 | 92.77 |
|  | A-2 | 1V1-2 | 0.250 | 39.6 | 454,721 | 130 | 115 | 92.69 |
|  | A-3 | 1V1-3 | 0.295 | 20.4 | 469,527 | 130 | 115 | 92.19 |
| Dec.  2013 | B-1 | 1V2-2 | 0.300 | 39.2 | 417,625 | 110 | 94 | 73.20 |
|  | B-2 | 1V2-3 | 0.295 | 53.1 | 517,039 | 142 | 131 | 73.67 |
|  | B-3 | 1V2-4 | 0.292 | 38.5 | 542,208 | 150 | 139 | 74.67 |
| July  2014 | C-1 | 2V1-1 | 0.301 | 13.9 | 362,499 | 100 | 89 | 92.38 |
|  | C-2 | 2V1-2 | 0.267 | 14.2 | 501,511 | 138 | 123 | 92.53 |
|  | C-3 | 2V1-3 | 0.291 | 14.4 | 432,207 | 119 | 107 | 92.12 |
| Aug.  2014 | D-1 | 2V2-1 | 0.295 | 17.2 | 489,336 | 138 | 132 | 95.12 |
|  | D-2 | 2V2-2 | 0.291 | 18.6 | 280,161 | 77 | 74 | 95.33 |
|  | D-3 | 2V2-3 | 0.294 | 21.0 | 351,407 | 971 | 935 | 95.3 |
| Oct.  2014 | E-1 | 3V1-1 | 0.294 | 6.19 | 363,382 | 981 | 914 | 91.30 |
|  | E-2 | 3V1-2 | 0.280 | 5.57 | 434,111 | 117 | 108 | 91.18 |
|  | E-3 | 3V1-3 | 0.290 | 7.22 | 472,732 | 135 | 124 | 91.47 |
| Nov.  2014 | F-1 | 3V2-1 | 0.294 | 7.29 | 444,056 | 122 | 114 | 91.14 |
|  | F-2 | 3V2-2 | 0.285 | 6.64 | 500,636 | 136 | 128 | 91.29 |
|  | F-3 | 3V2-3 | 0.292 | 5.53 | 323,376 | 883 | 794 | 91.66 |
