# Supplementary material for: Genome-resolved metagenomics of sugarcane vinasse bacteria
Source: Biotechnol Biofuels. 2018 Feb 22;11:48. doi: 10.1186/s13068-018-1036-9 (PMC5822648; doi:10.1186/s13068-018-1036-9)
Supplement: Supplementary file 2 — Additional file 2. Primers and thermocycler conditions used in gene abundance analysis by real time qPCR of the vinasse samples. [file 13068_2018_1036_MOESM2_ESM.docx]

**Genome-resolved metagenomics of sugarcane vinasse bacteria**

Noriko A. Cassman^1^, Késia S. Lourenço^1,2^, Janaína B. do Carmo^3^, Heitor Cantarella^2^, Eiko E. Kuramae^1^

^1^Department of Microbial Ecology, Netherlands Institute of Ecology NIOO-KNAW, Wageningen, Netherlands

^2^Soils and Environmental Resources Center, Agronomic Institute of Campinas, P.O. Box 28, 13012-970, Campinas, SP, Brazil

^3^Environmental Science Department*,* Federal University of São Carlos, 18052-780, Sorocaba, SP, Brazil

Correspondence: EE Kuramae, Department of Microbial Ecology, Netherlands Institute of Ecology NIOO-KNAW, Wageningen, Netherlands. Email: [e.kuramae@nioo.knaw.nl](mailto:e.kuramae@nioo.knaw.nl)

**Additional file 2.** Primers and thermocycler conditions used in gene abundance analysis by real time qPCR of the vinasse samples.

| **Target gene** | **Primer** | **Primer Sequence** | **Size (bp)** | **12 μL of reaction** | **Thermal profile** |
| --- | --- | --- | --- | --- | --- |
| AOA *amoA*  AOB *amoA* | Arch-amoAF  Arch-amoAR | 5’-STAATGGTCTGGCTTAGACG-3’  5’-GCGGCCATCCATCTGTATGT-3’ | 635  491 | 6 μL of Sybrgreen Bioline SensiFAST SYBR non-rox mix, 0.125 μL of each primer (10 pmol), 1.75 μL of BSA  and 4 μL of DNA (3 ng).  6 μL of Sybrgreen Bioline SensiFAST SYBR non-rox mix, 0.125 μL of each primer (10 pmol) and 4 μL of DNA (3 ng). | 95°C-5 min.; 40x 95°C-10s, 64°C-10s, 72°C-20s  95°C-10min.; 40x 95°C-10s, 65°C-25s, |
|  | amoA1F  amoA2R | 5’-GGGGTTTCTACTGGTGGT-3’  5’-CCCCTCKGSAAAGCCTTCTTC-3’ |  |  |  |
| AOA *amoA* | Arch-amoAF  Arch-amoAR | 5’-STAATGGTCTGGCTTAGACG-3’  5’-GCGGCCATCCATCTGTATGT-3’ | 635 | 6 μL of Sybrgreen Bioline SensiFAST SYBR non-rox mix, 0.125 μL of each primer (10 pmol), 1.75 μL of BSA  and 4 μL of DNA (3 ng). | 95°C-5 min.; 40x 95°C-10s, 64°C-10s, 72°C-20s |
|  | amoA2R | 5’-CCCCTCKGSAAAGCCTTCTTC-3’ |  |  |  |
| *Nos*Z [3] | nosZ2F | 5’-CGCRACGGCAASAAGGTSMSSGT-3’ | 267 | 6 μL of Sybrgreen Bioline SensiFAST SYBR non-rox mix, 0.250 μL of each primer (10 pmol), 1.20 μL of BSA and 4 μL of DNA (1.25 ng). | 95°C-5 min.; 40x 95°C-10s,  64°C-10s, 72°C-20s |
|  | nosZ2R | 5’-CAKRTGCAKSGCRTGGCAGAA-3’ |  |  |  |
| *nir*K [4] | NirK876 | 5'-ATYGGCGGVAYGGCGA-3' | 165 | 6 μL of Sybrgreen Bioline SensiFAST SYBR non-rox mix, 0.250 μL of each primer (10 pmol), 1.50 μL of BSA and 4 μL of DNA (1.25 ng). | 95°C-5 min.; 40x 95°C-15s,  62°C-15s, 72°C-20s |
|  | NirK1040 | 5'-GCCTCGATCAGRTTRTGGTT-3' |  |  |  |
| *nir*S [5] | nirScd3aF | 5'-GTSAACGTSAAGGARACSGG-3' | 425 | 6 μL of Sybrgreen Bioline SensiFAST SYBR non-rox mix, 0.250 μL of each primer (10 pmol), 1.20 μL of BSA and 4 μL of DNA (1.25 ng). | 95°C-5 min.; 40x 95°C-10s,  63°C-10s, 72°C-20s |
|  | nirSR3cd | 5'-GASTTCGGRTGSGTCTTGA-3' |  |  |  |

1. Francis CA, Roberts KJ, Beman JM, Santoro AE & Oakley BB. Ubiquity and diversity of ammonia-oxidizing archaea in water columns and sediments of the ocean. Proc Natl Acad Sci USA. 2005;102:14683–88.

2. Rotthauwe JH, Witzel KP & Liesack W. The Ammonia monooxygenase structural gene amoA as a functional marker: molecular fine-Scale analysis of natural ammonia-oxidizing populations. Appl Environ Microbiol. 1997;63: 4704 12.

3. Henry S, Bru D, Stres B, Hallet S & Philippot L. Quantitative detection of the nosZ gene, encoding nitrous oxide reductase, and comparison of the abundances of 16S rRNA, *nar*G, *nir*K, and *nos*Z genes in soils. App. Environ Microbiol. 2006;72: 5181–89.

4. Henry S, Baudoin E, López-Gutiérrez JC, Martin-Laurent F, Brauman A & Philippot L. Quantification of denitrifying bacteria in soils by *nir*K gene targeted real-time PCR. J Microbiol Methods**.** 2004;59:327–35.

5. Throbäck IN, Enwall K, Jarvis A & Hallin S. Reassessing PCR primers targeting *nir*S, *nir*K and *nos*Z genes for community surveys of denitrifying bacteria with DGGE. FEMS Microbiol Ecol. 2004;49:401–17.
