# Supplementary material for: Genome-resolved metagenomics of sugarcane vinasse bacteria
Source: Biotechnol Biofuels. 2018 Feb 22;11:48. doi: 10.1186/s13068-018-1036-9 (PMC5822648; doi:10.1186/s13068-018-1036-9)
Supplement: Supplementary file 4 — Additional file 4. Data description of the merged vinasse metagenomes uploaded to MG-RAST. [file 13068_2018_1036_MOESM4_ESM.docx]

**Genome-resolved metagenomics of sugarcane vinasse bacteria**

Noriko A. Cassman^1^, Késia S. Lourenço^1,2^, Janaína B. do Carmo^3^, Heitor Cantarella^2^, Eiko E. Kuramae^1^

^1^Department of Microbial Ecology, Netherlands Institute of Ecology NIOO-KNAW, Wageningen, Netherlands

^2^Soils and Environmental Resources Center, Agronomic Institute of Campinas, P.O. Box 28, 13012-970, Campinas, SP, Brazil

^3^Environmental Science Department*,* Federal University of São Carlos, 18052-780, Sorocaba, SP, Brazil

Correspondence: EE Kuramae, Department of Microbial Ecology, Netherlands Institute of Ecology NIOO-KNAW, Wageningen, Netherlands. Email: [e.kuramae@nioo.knaw.nl](mailto:e.kuramae@nioo.knaw.nl)

**Additional file 4.** Data description of the merged vinasse metagenomes uploaded to MG-RAST.

| MG-RAST ID | Sample Date | Sample Name | Sample ID |  | Percent merged |  | # merged reads | Avg. merged read length | # merged bases (Mbp) |
| --- | --- | --- | --- | --- | --- | --- | --- | --- | --- |
| 4678764.3 | Nov. 2013 | A-1 | 1V1-1 |  | 87.98% |  | 236896 | 592,17 | 85 |
| 4678762.3 |  | A-2 | 1V1-2 |  | 88.72% |  | 230245 | 592,38 | 83 |
| 4678758.3 |  | A-3 | 1V1-3 |  | 87.40% |  | 245213 | 590,59 | 83 |
| 4678765.3 | Dec. 2013 | B-1 | 1V2-2 |  | 93.07% |  | 263198 | 582,17 | 86 |
| 4678752.3 |  | B-2 | 1V2-3 |  | 95.62% |  | 359330 | 580,67 | 116 |
| 4678749.3 |  | B-3 | 1V2-4 |  | 95.15% |  | 376765 | 581,71 | 124 |
| 4678755.3 | July 2014 | C-1 | 2V1-1 |  | 88.74% |  | 193318 | 590,16 | 64 |
| 4678760.3 |  | C-2 | 2V1-2 |  | 91.90% |  | 275242 | 589,16 | 91 |
| 4678754.3 |  | C-3 | 2V1-3 |  | 91.96% |  | 252621 | 588,15 | 83 |
| 4678766.3 | Aug. 2014 | D-1 | 2V2-1 |  | 95.97% |  | 402629 | 581,89 | 139 |
| 4678761.3 |  | D-2 | 2V2-2 |  | 95.88% |  | 235413 | 577,50 | 79 |
| 4678753.3 |  | D-3 | 2V2-3 |  | 96.49% |  | 300148 | 576,57 | 100 |
| 4678756.3 | Oct. 2014 | E-1 | 3V1-1 |  | 94.29% |  | 258649 | 584,54 | 84 |
| 4678751.3 |  | E-2 | 3V1-2 |  | 92.39% |  | 293786 | 585,17 | 96 |
| 4678759.3 |  | E-3 | 3V1-3 |  | 91.44% |  | 305881 | 589,63 | 111 |
| 4678757.3 | Nov. 2014 | F-1 | 3V2-1 |  | 93.26% |  | 320201 | 586,67 | 109 |
| 4678763.3 |  | F-2 | 3V2-2 |  | 95.37% |  | 383266 | 583,43 | 126 |
| 4678750.3 |  | F-3 | 3V2-3 |  | 84.59% |  | 269124 | 575,92 | 94 |
