# Supplementary material for: Genome-resolved metagenomics of sugarcane vinasse bacteria
Source: Biotechnol Biofuels. 2018 Feb 22;11:48. doi: 10.1186/s13068-018-1036-9 (PMC5822648; doi:10.1186/s13068-018-1036-9)
Supplement: Supplementary file 5 — Additional file 5. Taxonomic distribution of the merged vinasse metagenomes from MG-RAST annotation against RefSeq database. Phyla with average relative abundance greater than 1% across all samples were included. Samples with significantly different phyla between groups (Tukey–Kramer post hoc test, 95% confidence interval, p < 0.001) are indicated by different letters. [file 13068_2018_1036_MOESM5_ESM.docx]

**Genome-resolved metagenomics of sugarcane vinasse bacteria**

Noriko A. Cassman^1^, Késia S. Lourenço^1,2^, Janaína B. do Carmo^3^, Heitor Cantarella^2^, Eiko E. Kuramae^1^

^1^Department of Microbial Ecology, Netherlands Institute of Ecology NIOO-KNAW, Wageningen, Netherlands

^2^Soils and Environmental Resources Center, Agronomic Institute of Campinas, P.O. Box 28, 13012-970, Campinas, SP, Brazil

^3^Environmental Science Department*,* Federal University of São Carlos, 18052-780, Sorocaba, SP, Brazil

Correspondence: EE Kuramae, Department of Microbial Ecology, Netherlands Institute of Ecology NIOO-KNAW, Wageningen, Netherlands. Email: [e.kuramae@nioo.knaw.nl](mailto:e.kuramae@nioo.knaw.nl)

**Additional file 5.** Taxonomic distribution of the merged vinasse metagenomes from MG-RAST annotation against RefSeq database. Phyla with average relative abundance greater than 1% across all samples were included. Samples with significantly different phyla between groups (Tukey-Kramer post-hoc test, 95% confidence interval, *p* < 0.001) are indicated by different letters.

|  |  |  | Average proportion of sample | | | | | |
| --- | --- | --- | --- | --- | --- | --- | --- | --- |
| Phylum | p-values (corrected) | Effect size | A | B | C | D | E | F |
| Firmicutes | 1.94E-14 | 0.998 | 44.5±0.4 a | 39.2±1.7 b | 61.2±0.9 c | 97.0±0.0 d | 58.8±0.5 e | 35.4±0.3 f |
| Bacteroidetes | 4.09E-14 | 0.998 | 29.4±1.5 a | 9.5±0.2 b | 11.3±0.7 c | 0.8±0.0 d | 11.5±0.3 e | 52.8±1.1 f |
| Actinobacteria | 1.88E-09 | 0.985 | 15.9±1.5 a | 2.1±0.1 b | 17.5±1.4 a | 0.4±0.0 b | 17.8±0.6 a | 3.3±0.8 b |
| Proteobacteria | 7.19E-14 | 0.997 | 0.8±0.0 a | 39.4±1.8 b | 0.8±0.0 a | 0.3±0.0 a | 1.7±0.0 a | 1.4±0.0 a |

Effect sizes and corrected p-values were calculated using ANOVA on mean relative abundance of phyla in sample groups using the Benjamini-Hochberg multiple test correction in STAMP.
