# Supplementary material for: Genome-resolved metagenomics of sugarcane vinasse bacteria
Source: Biotechnol Biofuels. 2018 Feb 22;11:48. doi: 10.1186/s13068-018-1036-9 (PMC5822648; doi:10.1186/s13068-018-1036-9)
Supplement: Supplementary file 6 — Additional file 6. Phylogenetic relationships between full-length 16S rRNA genes reconstructed from the vinasse metagenomes using REAGO. The 16S rRNA sequences from vinasse and reference sequences were aligned using ClustalW. The neighbor-joining tree was created with MEGA and visualized using iTol ignoring branch lengths. [file 13068_2018_1036_MOESM6_ESM.docx]

**Genome-resolved metagenomics of sugarcane vinasse bacteria**

Noriko A. Cassman^1^, Késia S. Lourenço^1,2^, Janaína B. do Carmo^3^, Heitor Cantarella^2^, Eiko E. Kuramae^1^

^1^Department of Microbial Ecology, Netherlands Institute of Ecology NIOO-KNAW, Wageningen, Netherlands

^2^Soils and Environmental Resources Center, Agronomic Institute of Campinas, P.O. Box 28, 13012-970, Campinas, SP, Brazil

^3^Environmental Science Department*,* Federal University of São Carlos, 18052-780, Sorocaba, SP, Brazil

Correspondence: EE Kuramae, Department of Microbial Ecology, Netherlands Institute of Ecology NIOO-KNAW, Wageningen, Netherlands. Email: [e.kuramae@nioo.knaw.nl](mailto:e.kuramae@nioo.knaw.nl)


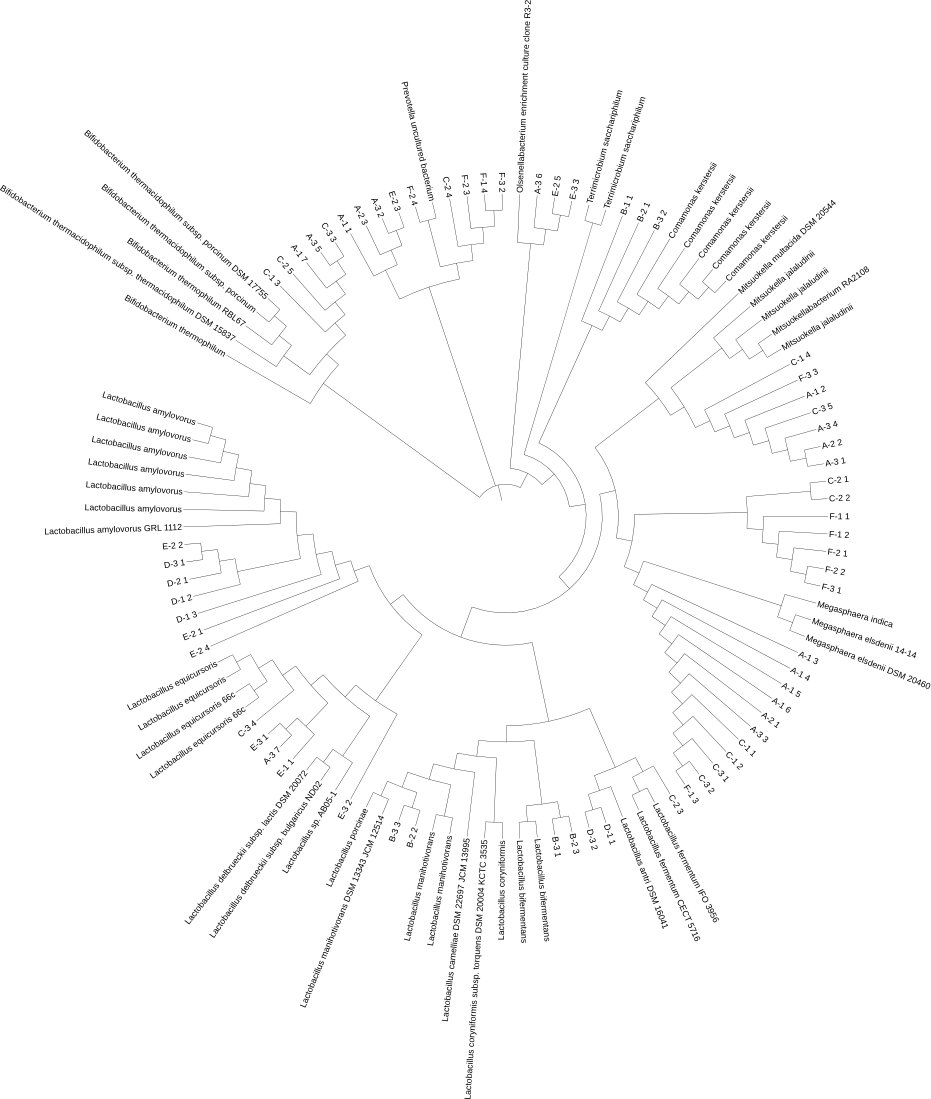


**Additional file 6.** Phylogenetic relationships between full-length 16S rRNA genes reconstructed from the vinasse metagenomes using REAGO. The 16S rRNA sequences from vinasse and reference sequences were aligned using ClustalW. The neighbor-joining tree was created with MEGA and visualized using iTol ignoring branch lengths.
