# Supplementary material for: Genome-resolved metagenomics of sugarcane vinasse bacteria
Source: Biotechnol Biofuels. 2018 Feb 22;11:48. doi: 10.1186/s13068-018-1036-9 (PMC5822648; doi:10.1186/s13068-018-1036-9)
Supplement: Supplementary file 7 — Additional file 7. Functional potential characterization of the vinasse samples from MG-RAST annotation against the Subsystems database. Only the subsystems at level 1 with average relative abundance greater than 2% across all samples were included. Significantly different subsystems at level 1 between sample groups (Tukey–Kramer post hoc test, 95% confidence interval, p < 0.05) are indicated by different letters. [file 13068_2018_1036_MOESM7_ESM.docx]

**Genome-resolved metagenomics of sugarcane vinasse bacteria**

Noriko A. Cassman^1^, Késia S. Lourenço^1,2^, Janaína B. do Carmo^3^, Heitor Cantarella^2^, Eiko E. Kuramae^1^

^1^Department of Microbial Ecology, Netherlands Institute of Ecology NIOO-KNAW, Wageningen, Netherlands

^2^Soils and Environmental Resources Center, Agronomic Institute of Campinas, P.O. Box 28, 13012-970, Campinas, SP, Brazil

^3^Environmental Science Department*,* Federal University of São Carlos, 18052-780, Sorocaba, SP, Brazil

Correspondence: EE Kuramae, Department of Microbial Ecology, Netherlands Institute of Ecology NIOO-KNAW, Wageningen, Netherlands. Email: [e.kuramae@nioo.knaw.nl](mailto:e.kuramae@nioo.knaw.nl)

Additional file 7. Comparison of genus abundances across samples from the A) MGRAST, B) extracted 16S and the C) bin taxonomy results. The colors correspond to genus and phyla as Phylum Firmicutes (green), Proteobacteria (red), Actinobacteria (brown) and Bacteroidetes (orange).

| A) |  |
| --- | --- |
| B) |  |
| C) |  |
