# Supplementary material for: Genome-resolved metagenomics of sugarcane vinasse bacteria
Source: Biotechnol Biofuels. 2018 Feb 22;11:48. doi: 10.1186/s13068-018-1036-9 (PMC5822648; doi:10.1186/s13068-018-1036-9)
Supplement: Supplementary file 9 — Additional file 9. Functional potential profiles of the top 30 pathways across the vinasse samples, excluding “unmapped” and “uncategorized” results. The functional group and sample profiles were clustered using hclust2 from humann2 analysis against the UniRef90 database. [file 13068_2018_1036_MOESM9_ESM.docx]

**Genome-resolved metagenomics of sugarcane vinasse bacteria**

Noriko A. Cassman^1^, Késia S. Lourenço^1,2^, Janaína B. do Carmo^3^, Heitor Cantarella^2^, Eiko E. Kuramae^1^

^1^Department of Microbial Ecology, Netherlands Institute of Ecology NIOO-KNAW, Wageningen, Netherlands

^2^Soils and Environmental Resources Center, Agronomic Institute of Campinas, P.O. Box 28, 13012-970, Campinas, SP, Brazil

^3^Environmental Science Department*,* Federal University of São Carlos, 18052-780, Sorocaba, SP, Brazil

Correspondence: EE Kuramae, Department of Microbial Ecology, Netherlands Institute of Ecology NIOO-KNAW, Wageningen, Netherlands. Email: [e.kuramae@nioo.knaw.nl](mailto:e.kuramae@nioo.knaw.nl)

**Additional file 9.** Functional potential characterization of the vinasse metagenomes from MG-RAST annotation against the Subsystems database**.** Only the subsystems at level 1 with average relative abundance greater than 2% across all samples were included. Significantly different subsystems at level 1 between sample groups (Tukey-Kramer post-hoc test, 95% confidence interval, *p* < 0.05) are indicated by different letters.

|  | | | Relative sample abundance | | | | | |
| --- | --- | --- | --- | --- | --- | --- | --- | --- |
| Subsystems Level 1 Category | p-values (corrected) | Effect size | A | B | C | D | E | F |
| Carbohydrates | 2.84E-08 | 0.967 | 16.3±0.3ac | 12.9±0.2b | 15.5±0.1a | 15.5±0.1a | 13.7±0.3b | 17.0±0.5c |
| Clustering-based subsystems | 4.37E-09 | 0.977 | 14.5±0.2a | 14.6±0.0a | 15.2±0.1b | 17.2±0.0c | 14.8±0.2ab | 14.2±0.2a |
| Amino Acids and Derivatives | 6.35E-13 | 0.997 | 9.2±0.1 ac | 9.4±0.0 a | 9.3±0.1 a | 4.7±0.1 b | 7.0±0.2 d | 8.9±0.0 c |
| Miscellaneous | 8.50E-05 | 0.863 | 7.6±0.1 a | 7.5±0.0 a | 7.6±0.1 a | 7.3±0.0 b | 7.0±0.2 c | 7.3±0.1 bc |
| Protein Metabolism | 2.25E-08 | 0.969 | 7.1±0.1 a | 6.9±0.1 a | 7.8±0.1 c | 8.3±0.1 d | 7.0±0.1 ab | 6.7±0.2 b |
| DNA Metabolism | 3.86E-11 | 0.991 | 5.5±0.1 a | 5.4±0.0 a | 5.4±0.1 a | 7.3±0.1 c | 5.8±0.0 b | 5.7±0.0 b |
| RNA Metabolism | 3.43E-07 | 0.949 | 5.7±0.1 a | 5.7±0.1 a | 5.9±0.1 a | 7.0±0.1 c | 5.7±0.2 a | 5.1±0.1 b |
| Cofactors, Vitamins | 3.71E-11 | 0.991 | 5.6±0.0 a | 5.8±0.1 a | 5.1±0.1 b | 3.5±0.0 c | 4.0±0.2 d | 5.2±0.0 b |
| Cell Wall and Capsule | 3.04E-06 | 0.923 | 4.7±0.1 ac | 4.4±0.1 bc | 4.6±0.1 c | 4.9±0.1 a | 4.2±0.1 b | 4.9±0.0 a |
| Phages and Prophages | 8.82E-08 | 0.960 | 2.9±0.1 a | 2.3±0.0 a | 3.1±0.0 a | 2.4±0.0 a | 10.0±1.4 b | 2.6±0.1 a |
| Nucleosides and Nucleotides | 4.42E-10 | 0.984 | 3.2±0.0 a | 2.7±0.0 b | 3.3±0.0 a | 3.7±0.1 c | 4.1±0.1 d | 2.9±0.1 b |

Effect sizes and corrected p-values were calculated using ANOVA on mean relative abundance of Subsystems level 1 in sample groups using the Benjamini-Hochberg multiple test correction in STAMP.
