# Supplementary material for: Genome-resolved metagenomics of sugarcane vinasse bacteria
Source: Biotechnol Biofuels. 2018 Feb 22;11:48. doi: 10.1186/s13068-018-1036-9 (PMC5822648; doi:10.1186/s13068-018-1036-9)
Supplement: Supplementary file 10 — Additional file 10. All vinasse bin characteristics and relative sample abundances (indicated by heatmap per sample). Bin id’s highlighted in green indicate “good” bins; yellow id’s indicate “interesting” bins, and red indicate “bad” bins. [file 13068_2018_1036_MOESM10_ESM.docx]

**Genome-resolved metagenomics of sugarcane vinasse bacteria**

Noriko A. Cassman^1^, Késia S. Lourenço^1,2^, Janaína B. do Carmo^3^, Heitor Cantarella^2^, Eiko E. Kuramae^1^

^1^Department of Microbial Ecology, Netherlands Institute of Ecology NIOO-KNAW, Wageningen, Netherlands

^2^Soils and Environmental Resources Center, Agronomic Institute of Campinas, P.O. Box 28, 13012-970, Campinas, SP, Brazil

^3^Environmental Science Department*,* Federal University of São Carlos, 18052-780, Sorocaba, SP, Brazil

Correspondence: EE Kuramae, Department of Microbial Ecology, Netherlands Institute of Ecology NIOO-KNAW, Wageningen, Netherlands. Email: [e.kuramae@nioo.knaw.nl](mailto:e.kuramae@nioo.knaw.nl)


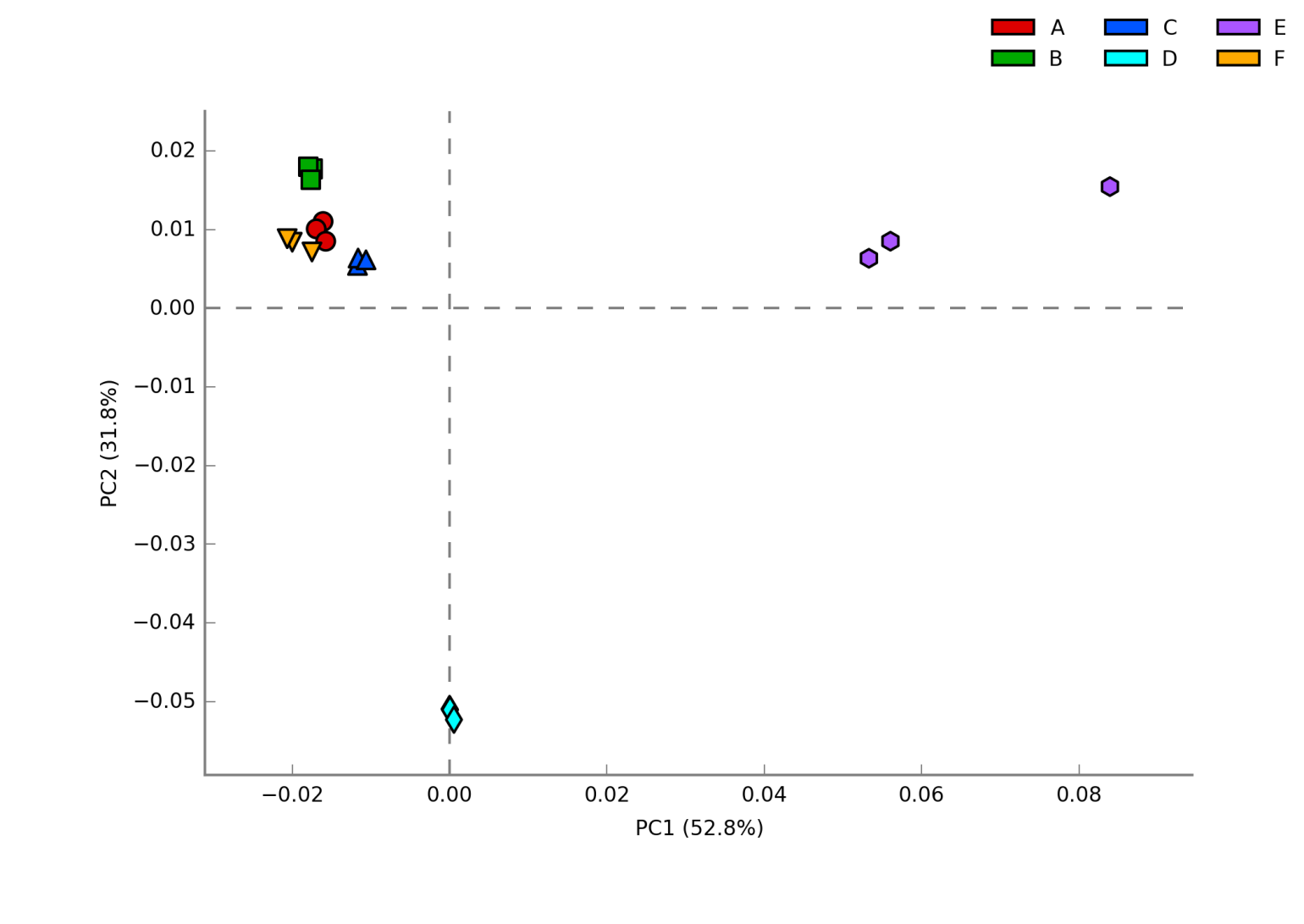


**Additional file 10.** Principal component analysis of the Subsystems Level 1 category abundances of the vinasse metagenomes. The colors correspond to time point. Profiles were determined against the Subsystems database using MG-RAST and relative abundances of phyla were calculated out of the total number of sample reads.
