# Supplementary material for: Genome-resolved metagenomics of sugarcane vinasse bacteria
Source: Biotechnol Biofuels. 2018 Feb 22;11:48. doi: 10.1186/s13068-018-1036-9 (PMC5822648; doi:10.1186/s13068-018-1036-9)
Supplement: Supplementary file 12 — Additional file 12. All vinasse bin taxonomic affiliations based on CAT. Bin id’s highlighted in green indicate “good” bins; yellow id’s indicate “interesting” bins, and red indicate “bad” bins. [file 13068_2018_1036_MOESM12_ESM.docx]

**Genome-resolved metagenomics of sugarcane vinasse bacteria**

Noriko A. Cassman^1^, Késia S. Lourenço^1,2^, Janaína B. do Carmo^3^, Heitor Cantarella^2^, Eiko E. Kuramae^1^

^1^Department of Microbial Ecology, Netherlands Institute of Ecology NIOO-KNAW, Wageningen, Netherlands

^2^Soils and Environmental Resources Center, Agronomic Institute of Campinas, P.O. Box 28, 13012-970, Campinas, SP, Brazil

^3^Environmental Science Department*,* Federal University of São Carlos, 18052-780, Sorocaba, SP, Brazil

Correspondence: EE Kuramae, Department of Microbial Ecology, Netherlands Institute of Ecology NIOO-KNAW, Wageningen, Netherlands. Email: [e.kuramae@nioo.knaw.nl](mailto:e.kuramae@nioo.knaw.nl)

**Additional file 12.** All vinasse bin characteristics and relative sample abundances (indicated by heatmap per sample). Bin id’s highlighted in green indicate “good” bins; yellow id’s indicate “interesting” bins, and red id’s indicate “bad” bins.

| **Bin**  **Id** | **A** | **B** | **C** | **D** | **E** | **F** | **Length**  **(Mbp)** | **# Contigs** | **N50** | **GC**  **(%)** | **Completeness**  **(%)** | **Redundancy**  **(%)** |
| --- | --- | --- | --- | --- | --- | --- | --- | --- | --- | --- | --- | --- |
| 1 | 6 | 1 | 2 | 0 | 0 | 1 | 2.4 | 209 | 23171 | 53 | 92 | 2 |
| 2 | 14 | 2 | 1 | 0 | 4 | 3 | 3.5 | 547 | 10519 | 49 | 94 | 4 |
| 3 | 8 | 1 | 18 | 0 | 2 | 13 | 2.2 | 352 | 10534 | 53 | 97 | 2 |
| 5 | 3 | 0 | 4 | 0 | 2 | 1 | 1.9 | 298 | 11078 | 60 | 94 | 3 |
| 6 | 4 | 1 | 2 | 0 | 1 | 12 | 2.4 | 539 | 6347 | 44 | 91 | 2 |
| 7 | 9 | 2 | 3 | 0 | 4 | 2 | 4.9 | 2521 | 1944 | 51 | 63 | 40 |
| 9 | 2 | 0 | 2 | 0 | 1 | 0 | 2.0 | 407 | 6384 | 60 | 90 | 6 |
| 10 | 1 | 1 | 1 | 1 | 3 | 0 | 2.1 | 183 | 27583 | 47 | 96 | 1 |
| 11 | 7 | 2 | 4 | 0 | 2 | 10 | 5.8 | 2606 | 2396 | 44 | 36 | 9 |
| 12 | 3 | 0 | 2 | 0 | 5 | 1 | 2.3 | 485 | 7489 | 66 | 91 | 5 |
| 13 | 2 | 0 | 2 | 0 | 0 | 0 | 1.5 | 605 | 2710 | 63 | 71 | 12 |
| 14 | 2 | 0 | 1 | 0 | 0 | 1 | 1.9 | 947 | 2190 | 52 | 74 | 15 |
| 15 | 2 | 0 | 2 | 0 | 1 | 1 | 2.2 | 1017 | 2297 | 53 | 76 | 9 |
| 16 | 1 | 1 | 1 | 0 | 0 | 13 | 3.0 | 307 | 22370 | 42 | 99 | 1 |
| 17 | 2 | 0 | 1 | 0 | 1 | 0 | 1.4 | 928 | 1434 | 60 | 43 | 14 |
| 18 | 2 | 0 | 1 | 0 | 2 | 0 | 1.5 | 893 | 1665 | 63 | 69 | 22 |
| 19 | 1 | 0 | 1 | 0 | 3 | 0 | 2.0 | 917 | 2351 | 62 | 60 | 12 |
| 20 | 0 | 0 | 1 | 0 | 1 | 1 | 1.2 | 387 | 3766 | 54 | 64 | 2 |
| 21 | 0 | 0 | 1 | 1 | 2 | 1 | 1.8 | 298 | 9992 | 50 | 88 | 1 |
| 22 | 2 | 1 | 3 | 0 | 1 | 12 | 5.3 | 2375 | 2491 | 36 | 39 | 7 |
| 23 | 0 | 1 | 1 | 3 | 2 | 1 | 1.9 | 373 | 7041 | 47 | 95 | 4 |
| 24 | 0 | 2 | 1 | 0 | 1 | 0 | 1.8 | 220 | 13204 | 53 | 98 | 4 |
| 25 | 1 | 1 | 1 | 1 | 0 | 2 | 1.8 | 729 | 2822 | 40 | 91 | 9 |
| 26 | 1 | 1 | 2 | 0 | 1 | 1 | 2.1 | 1236 | 1733 | 41 | 66 | 15 |
| 27 | 0 | 1 | 1 | 45 | 3 | 1 | 1.9 | 262 | 11858 | 38 | 99 | 1 |
| 28 | 0 | 3 | 0 | 0 | 0 | 0 | 2.1 | 340 | 8670 | 38 | 96 | 5 |
| 29 | 0 | 1 | 0 | 2 | 0 | 0 | 1.0 | 439 | 2658 | 50 | 88 | 9 |
| 30 | 0 | 3 | 1 | 3 | 2 | 3 | 3.9 | 2021 | 1897 | 31 | 47 | 16 |
| 31 | 0 | 0 | 8 | 0 | 0 | 0 | 3.3 | 1595 | 2241 | 54 | 79 | 37 |
| 32 | 0 | 0 | 0 | 6 | 0 | 0 | 2.0 | 104 | 208993 | 36 | 99 | 1 |
| 33 | 0 | 0 | 0 | 1 | 3 | 0 | 1.7 | 447 | 4850 | 48 | 96 | 9 |
| 34 | 0 | 3 | 0 | 0 | 0 | 0 | 2.7 | 343 | 12289 | 60 | 92 | 1 |
| 35 | 0 | 4 | 0 | 0 | 0 | 0 | 2.7 | 259 | 13750 | 43 | 96 | 6 |
| 36 | 0 | 5 | 0 | 0 | 0 | 0 | 1.7 | 647 | 2989 | 49 | 67 | 5 |
| 36.2 | 0 | 4 | 0 | 0 | 0 | 0 | 1.4 | 868 | 1558 | 48 | 38 | 5 |
| 36.3 | 0 | 1 | 0 | 0 | 0 | 0 | 0.5 | 245 | 2503 | 48 | 10 | 0 |
| 37.1 | 0 | 10 | 0 | 0 | 0 | 0 | 3.1 | 1326 | 2603 | 57 | 77 | 24 |
| 37.2 | 0 | 4 | 0 | 0 | 0 | 0 | 1.2 | 784 | 1533 | 58 | 34 | 6 |
| 37.3 | 0 | 2 | 0 | 0 | 0 | 0 | 0.7 | 506 | 1236 | 49 | 16 | 1 |
| 38 | 0 | 9 | 0 | 0 | 0 | 0 | 3.0 | 488 | 9382 | 60 | 89 | 3 |
| 39 | 0 | 0 | 0 | 4 | 0 | 0 | 1.9 | 205 | 15510 | 47 | 99 | 4 |
| 39.2 | 0 | 0 | 0 | 0 | 0 | 0 | 0.1 | 76 | 1387 | 47 | 5 | 1 |
| 40.1 | 0 | 4 | 0 | 0 | 0 | 0 | 1.6 | 190 | 11919 | 28 | 97 | 2 |
| 40.2 | 0 | 2 | 0 | 0 | 0 | 0 | 0.6 | 271 | 2044 | 27 | 15 | 1 |
