# Supplementary material for: Genome-resolved metagenomics of sugarcane vinasse bacteria
Source: Biotechnol Biofuels. 2018 Feb 22;11:48. doi: 10.1186/s13068-018-1036-9 (PMC5822648; doi:10.1186/s13068-018-1036-9)
Supplement: Supplementary file 13 — Additional file 13. Principal component analysis of the phylum-level abundance distributions of the vinasse samples. Relative abundance profiles were determined using MG-RAST against the RefSeq database and phyla membership was determined using the Last Common Ancestor algorithm. [file 13068_2018_1036_MOESM13_ESM.docx]

**Genome-resolved metagenomics of sugarcane vinasse bacteria**

Noriko A. Cassman^1^, Késia S. Lourenço^1,2^, Janaína B. do Carmo^3^, Heitor Cantarella^2^, Eiko E. Kuramae^1^

^1^Department of Microbial Ecology, Netherlands Institute of Ecology NIOO-KNAW, Wageningen, Netherlands

^2^Soils and Environmental Resources Center, Agronomic Institute of Campinas, P.O. Box 28, 13012-970, Campinas, SP, Brazil

^3^Environmental Science Department*,* Federal University of São Carlos, 18052-780, Sorocaba, SP, Brazil

Correspondence: EE Kuramae, Department of Microbial Ecology, Netherlands Institute of Ecology NIOO-KNAW, Wageningen, Netherlands. Email: [e.kuramae@nioo.knaw.nl](mailto:e.kuramae@nioo.knaw.nl)

**Additional file 13.** All vinasse bin taxonomic affiliations based on CAT. Bin id’s highlighted in green indicate “good” bins; yellow id’s indicate “interesting” bins, and red indicate “bad” bins.

| Bin Id | Kingdom | Phylum | Class | Order | Family | Genus | Species |
| --- | --- | --- | --- | --- | --- | --- | --- |
| 1 | B | F | Nega | Selenomonadales | Veillonellaceae | Mitsuokella | Unclassified |
| 2 | B | B | Bact | Bacteroidales | Prevotellaceae | Prevotella | P. mutisaccharivorax/Unclassified |
| 3 | B | F | Nega | Selenomonadales | Veillonellaceae | Megasphaera | Unclassified/M. elsdenii |
| 4.1 | V |  |  |  | Caudovirales | Siphovirales | Lactobacillus phage Ldl1 |
| 4.2 | B | U/B | U/B | U/B | U | U | U |
| 5 | B | A | Acti | Bifidobacteriales | Bifidobacteriaceae | Bifidobacterium | Unclassified |
| 6 | B | B | Bact | Bacteroidales | Prevotellaceae/Unclassified | Prevotella/Unclassified | Unclassified |
| 7 | B | A/B/F |  |  |  |  |  |
| 8.1 | B/V | U | U | U | U/Caudovirales | U/Siphoviridae | U/Lactobacillus phage LdL1 |
| 8.2 | B | F | B | Lactobacillales | Lactobacillaceae | Lactobacillus | U |
| 8.3 | B | B | Bact | Bacteroidales | Prevotellaceae | Prevotella | P. histicola/U |
| 8.4 | B | F | Bac | Lactobacillales | Lactobacillaceae | Lactobacillus | U |
| 8.5 | B | B | U/Bact |  |  |  |  |
| 8.6 | B | F | Bac | Lactobacillales | Lactobacillaceae | Lactobacillus | U |
| 9 | B | U/A |  |  |  |  |  |
| 10 | B | F | Bacilli | Lactobacillales | Lactobacillaceae | Lactobacillus | L. equicursoris/Unclassified |
| 11 | B | B/F/U |  |  |  |  |  |
| 12 | B | A/U |  |  |  |  |  |
| 13 | B | U/A |  |  |  |  |  |
| 14 | B | F | Nega | Selenomonadales | Veillonellaceae | Megasphaera | U/sp. DJF_B143 |
| 15 | B | F | Nega | Selenomonadales | Veillonellaceae | Dialister | U/D. succinatiphilus |
| 16 | B | B | Bact | Bacteroidales | Prevotellaceae | Prevotella | U |
| 17 | B | A/F |  |  |  |  |  |
| 18 | U/B |  |  |  |  |  |  |
| 19 | B | U/A |  |  |  |  |  |
| 20 | B | F | Clos | Clostridiales | Eubacteriaceae/U | Pseudoramibacter/U | U/P. alactolyticus |
| 21 | B | F | Bacilli | Lactobacillales | Lactobacillaceae | Lactobacillus | L. delbrueckii/U |
| 22 | B | B/F |  |  |  |  |  |
| 23 | B | F | Bacilli | Lactobacillales | Lactobacillaceae | Lactobacillus | L. mucosae/U |
| 24 | B | F | Bacilli | Lactobacillales | Lactobacillaceae | Lactobacillus | L. fermentum/U |
| 25 | B | F | Bacilli | Lactobacillales | Lactobacillaceae | Lactobacillus | U/L. vini |
| 26 | B | F | Bacilli | Lactobacillales | Lactobacillaceae | Lactobacillus | U/L. agilis |
| 27 | B | F | Bacilli | Lactobacillales | Lactobacillaceae | Lactobacillus | U |
| 28 | B | F | Bacilli | Lactobacillales | Lactobacillaceae | Lactobacillus | L. vini |
| 29 | B | F | Clos | Clostridiales | Clostridiaceae | Clostridium | sp. CAG:568/U |
| 30 | B/A | F/E | Bacilli/Metha | Bacteroidales/Methanobacteriaceae | Lactobacillaceae/Methanobactericeae | Lactobactillus/Methanobrevibacter | U |
| 31 | B | F | Nega | Selenomonadales | Veillonellaceae | Megasphaera/U | U |
| 32 | B | F | Nega | Lactobacillales | L | L | U |
| 33 | B | F | B | Lactobacillales | L | L | L. secaliphilus/U |
| 34 | B | F | B | Lactobacillales | L | L | U/L. manihotivorans |
| 35 | B | F | B | Lactobacillales | L | L | L. bifermentans/U |
| 36 | B | P | Beta | Burkholderiales | Alcaligenaceae | U | U |
| 36.2 | B | P/F |  |  |  |  |  |
| 36.3 | B | F | B | Lactobacillales | L | L | U/L. manihotivorans |
| 37.1 | B | P | B | Burkholderiales | Alcaligenaceae | Alcaligenes | A. faecalis/U |
| 37.2 | B | P | B | B | A | Alcaligenes | U/A. faecalis |
| 37.3 | B/A | P/E |  |  |  |  |  |
| 38 | B | P | B | B | Comamonadaceae | U/Comamonas | U/C. kerstersii |
| 39 | B | F | B | Lactobacillales | L | L | U/L. panis |
| 39.2 | B | F | B | Lactobacillales | L | L | L. panis/L. ponis/U |
| 40.1 | B | P | E | Campylobacterales | Campylobacteraceae | Arcobacter | A. skirrowii/U |
| 40.2 | B/A | P/E | E/Metha | Campylobacterales/Methanobacteriales | Campylobacteraceae/Methanobacteriaceae | Arcobacter/Methanobrevibacter | U |

F = *Firmicutes*, B = *Bacteroidetes*, A = *Actinobacteria*, P = *Proteobacteria*, E = Euryarchaeota, U = Unknown, Bact = *Bacteroidia*, Nega = *Negativicutes*, Clos = *Clostridia*, Mega = *Megasphaera*, Lact = *Lactobacillales*
